# Morphological and molecular identification of symphylans (Myriapoda, Symphyla) from Colombian pineapple crops, with descriptions of two new species

**DOI:** 10.3897/zookeys.1268.159237

**Published:** 2026-02-10

**Authors:** José Mauricio Montes-Rodríguez, Claudia M. Holguin, Antonio Parra-Gómez, Sergio Marchant

**Affiliations:** 1 Corporación Colombiana de Investigación Agropecuaria - AGROSAVIA, Centro de Investigación La Suiza, Km 32 vía al mar, vereda Galápagos, Rionegro, Santander, Colombia Universidad Industrial de Santander Bucaramanga Colombia; 2 Facultad de Ciencias, Universidad Austral de Chile, Av. Rector Eduardo Morales Miranda 23, Valdivia, Chile Universidad Austral de Chile Valdivia Chile; 3 Universidad Industrial de Santander, Escuela de Biología, Laboratorio de Fisiología, Genómica y Transcripómica, Cra 27 calle 9 Edificio Laboratorios de Livianos, Bucaramanga, Santander, Colombia Corporación Colombiana de Investigación Agropecuaria - AGROSAVIA, Centro de Investigación La Suiza Rionegro Colombia

**Keywords:** Cytochrome oxidase I (COI), DNA barcode, *
Hanseniella
*, soil-dwelling arthropod, species delimitation, subterranean pest, *
Symphylella
*, taxonomy

## Abstract

Symphylans are soil-dwelling arthropods that can cause significant damage to agricultural crops, particularly pineapple. Despite their economic importance, their taxonomy and biodiversity remain poorly understood in Colombia, and the Neotropics. Here the symphylan species associated with pineapple crops were investigated in Santander, Colombia, the country’s largest pineapple-producing region. Symphylans were sampled from four commercial pineapple fields using baited pitfall traps. Morphological examination and DNA barcoding of the mitochondrial cytochrome c oxidase subunit I (COI) gene were used to identify the collected specimens. Six symphylan morphospecies were identified, including four *Hanseniella* and two *Symphylella*. The molecular analysis revealed four distinct genetic clades within the sequenced specimens. The integration of morphological and molecular data resolved initial taxonomic uncertainties, indicating that some previously separated morphospecies represent intraspecific morphological variation. Our results conclude that *Hanseniella cf. unguiculata* is the predominant species in pineapple crops, accounting for 95.9% of records. Additionally, two new species are described: *Hanseniella chocoita***sp. nov**. and *Hanseniella lebrijana***sp. nov**. A revised dichotomous key for the identification for *Hanseniella* species present in South America is also provided. This study provides valuable insights into the symphylan species inhabiting Colombian pineapple crops and emphasizes the need for further research to fully understand their diversity and evolutionary relationships.

## Introduction

Symphylans (Myriapoda, Symphyla) are soil-dwelling arthropods recognized as significant agricultural pests, particularly in pineapple [*Ananas comosus* (L.) Merr. (Bromeliaceae)], one of the world’s most important tropical fruits (Montes-Rodríguez and Ossa-Yepes 2021). In Colombia, the department of Santander stands as the nation’s leading pineapple producer (UPRA 2023), making the health of this crop vital to the regional economy. Symphylan feeding activity inflicts direct damage on the root systems of pineapple plants, creating stunted and inefficient roots that impair nutrient and water uptake, ultimately delaying plant growth and reducing fruit yield and quality (Rohrbach and Johnson 2003). Furthermore, the feeding wounds serve as entry points for opportunistic soil-borne pathogens like *Fusarium* spp., *Rhizoctonia* spp., and *Phytophthora* spp., which can lead to severe root rot and plant death (Saavedra 1990). Despite their clear economic impact, fundamental knowledge of symphylan taxonomy, diversity, and ecology in the agricultural landscapes of the Neotropics remains remarkably sparse (Scheller 1992; Parra-Gómez et al. 2024; Porta et al. 2024).

Effective and sustainable pest management strategies are fundamentally dependent on the accurate identification of the pest species involved. However, the taxonomic foundation for Neotropical symphylans is far from complete. While the monographic work of Hansen (1903) established the basis for symphylan systematics, much of the subsequent research has focused on other global regions (e.g., Edwards 1959; Scheller 1961; Scheller 1986; Domínguez and Vandenspiegel 2012). For the Neotropics, foundational contributions by Scheller (1992) and Scheller and Adis (1996, 2002) provided keys and species lists, primarily focused on the Amazon basin, leaving vast areas like the Andean agricultural regions poorly explored. Early reports from Colombia identified *Scutigerella immaculata* (Newport, 1845) as the primary symphylan pest in pineapple (Agredo et al. 1988). However, the later description of *Hanseniella colombiana* from the country (Juberthie-Jupeau and Réveillet 1997) highlighted the likelihood of a more complex and unresolved diversity. Subsequently, symphylan pests from flower crops in Colombia were identified; however, the COI sequences obtained unexpectedly matched only previously sequenced specimens from Cameroon available in public databases (Salazar-Moncada et al. 2015). These findings highlight a broader taxonomic uncertainty that limits our understanding of which species are responsible for crop damage and constrains the development of targeted control strategies.

This uncertainty is also compounded by significant methodological hurdles. Species-level identification has traditionally relied on subtle morphological characters, such as the arrangement of setae (chaetotaxy) and morphometric ratios (e.g., Hansen 1903; Scheller and Adis 2002; Domínguez and Vandenspiegel 2012), which are difficult to observe on these small, edaphic, whitish arthropods (Domínguez 2015). These difficulties are particularly pronounced in species-rich and morphologically conserved genera like *Hanseniella*, which, with more than 80 described species, presents a formidable challenge for identification based on morphology alone (see Appendix in Soesbergen 2019). To overcome these limitations, an integrative approach combining classical taxonomy with molecular tools is essential. DNA barcoding, utilizing the mitochondrial cytochrome c oxidase subunit I (COI) gene, has proven to be a powerful tool for species delimitation and identification in various arthropod groups, including symphylans in Colombia and China (Salazar-Moncada et al. 2015; Jin et al. 2023). This molecular approach can help resolve taxonomic ambiguities, uncover cryptic species, and provide robust data for phylogenetic analysis.

Given the economic importance of pineapple production in Santander, the unresolved taxonomy of pest symphylans, and the inherent challenges of morphological identification, in this study we aimed to conduct the first comprehensive survey of symphylan species associated with pineapple crops in this key agricultural region of Colombia. By employing an integrative taxonomic framework that combines detailed morphological analysis with DNA barcoding, we sought to identify the symphylan species present and establish a foundational dataset that can guide the development of effective, species-specific pest management strategies for the Colombian pineapple industry.

## Materials and methods

### Sample collection and preservation

**Figure 1.**
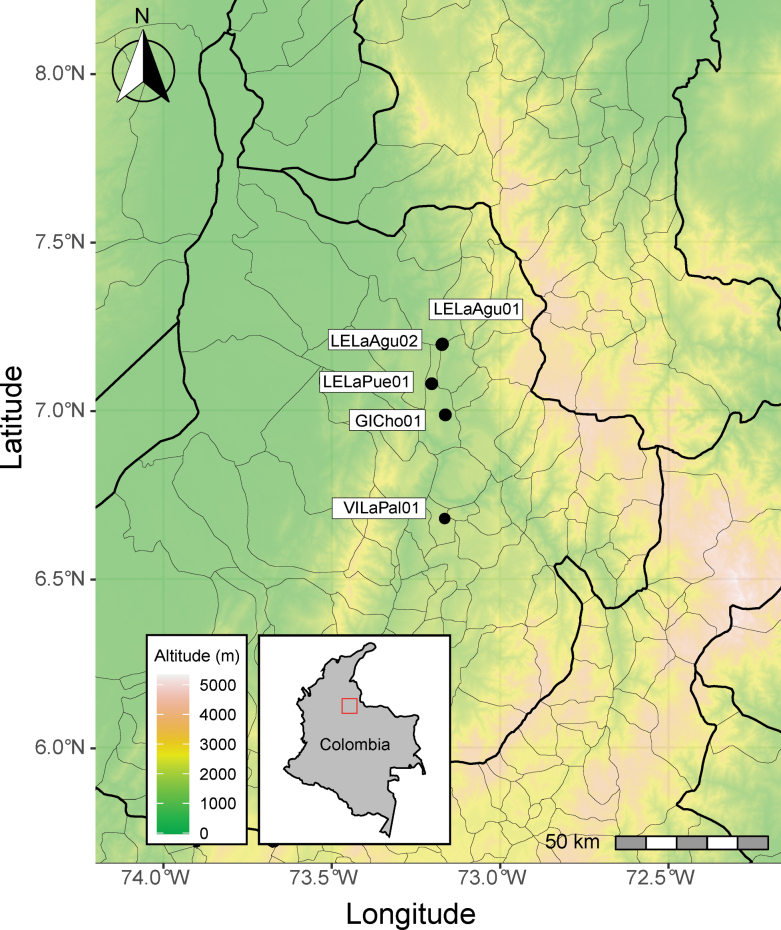
Distribution of symphylan sampling sites in pineapple crops across Santander, Colombia. Black dots indicate the locations of pineapple fields where symphylans were sampled. Department and municipality boundaries are delineated by thick and thin black lines, respectively. Base map and data adapted from OpenStreetMap and OpenStreetMap Foundation (©OpenStreetMap contributors).

This study investigated symphylan populations associated with commercial pineapple fields in Santander, Colombia, the country’s leading pineapple-producing region. Sampling was conducted in four localities across the region, representing a range of environmental conditions and management practices (Fig. 1, Table 1). We specifically targeted pineapple fields that were at least six months old to ensure the establishment of symphylan populations.

**Table 1.** Characteristics of pineapple farms sampled for symphylans in Santander, Colombia.

Municipality	Rural district / farm code	Coordinates (Latitude, Longitude)	Altitude (m)	Pineapple variety	No. of traps	Sampling date (month–year)
Lebrija	La Aguada / LELaAgu02	7.1950, -73.173	850	Perolera	40	IX, XI–2019
Lebrija	La Aguada / LELaAgu01	7.1978, -73.1714	975	MD-2	40	IX, XI–2019
Lebrija	La Puente / LELaPue01	7.0790, -73.2033	1300	Perolera × MD-2	40	IX, XI–2019
Villanueva	La Palma / VLaPal01	6.6790, -73.1647	1500	MD-2	20	III–2020
Girón	Chocoita / GICho01	6.9867, -73.1628	850	MD-2	40	VIII, IX–2020

To capture symphylans, we employed a modified version of the trapping method described by Soler et al. (2011), later adapted by Montes-Rodríguez and Ossa-Yepes (2021), which has proven effective for symphylan sampling in tropical agricultural systems. This method involved using 250 ml plastic containers with perforations to allow the entry of symphylan individuals. Each container was baited with 50 g of pieces of raw potato mixed with soil from the respective field, providing a food source attractive to symphylans. The containers were buried in the pineapple fields at a depth of ~ 10 cm. Traps were arranged in a randomized block design within each field to ensure representative sampling. The number of traps deployed varied across sampling sites, ranging from 20–40, depending on the field size and accessibility (Table 1).

After a three-day trapping period, the containers were carefully retrieved and transported to the entomology laboratory at the Colombian Corporation for Agricultural Research - Agrosavia - La Suiza research center for processing. In the laboratory, the contents of each trap (soil and bait) were emptied onto plastic trays and meticulously examined under a dissecting microscope to extract symphylans. Collected specimens were then gently transferred using fine-tipped forceps to individually labelled vials containing 70% ethyl alcohol for preservation and subsequent morphological and molecular analyses. All arthropod collection activities were conducted under the authority of collecting permit 1466 of 2014, granted by the National Authority of Environmental Licenses (ANLA). Voucher specimens and type material of the identified symphylan species were deposited in the Colección Taxonómica Nacional de Insectos “Luís María Murillo” (CTNI), Bogotá, Colombia.

### Morphological identification

Symphylan specimens were initially identified to the genus level using the taxonomic key for the Neotropical region provided by Scheller and Adis (2002). For detailed morphological examination and species-level identification, a representative subset of 50 individuals, encompassing the observed morphological variability, was selected for slide mounting. Following established slide-mounting techniques for mealybugs (Kondo and Watson 2022), specimens were carefully prepared to preserve morphological details. The number of specimens reported in the morphological character tables (Tables 3, 4) represents the final, curated subset of high-quality, slide-mounted individuals suitable for detailed examination.

A total of 15 morphological characters, primarily focusing on chaetotaxy and length/width ratios, were examined for each specimen. These characters were selected based on their diagnostic value in previous taxonomic studies of *Hanseniella* and *Symphylella* (Hansen 1903; Adam and Burtel 1956; Edwards 1959; Scheller 1961; Scheller 1971; Scheller 1986; Scheller 2007; Domínguez and Vandenspiegel 2012; Soesbergen 2019). To maximize the use of collected specimens for both morphological and molecular analyses, a second selection of individuals was made. Each specimen was carefully divided into three sections: the anterior portion, the posterior portion, and the middle third of the body. The anterior and posterior sections, which contain most of the taxonomically informative characters, were slide-mounted on Hoyer's medium for detailed examination under a compound microscope. The middle third of the body, deemed to have limited taxonomic value, was reserved for DNA extraction and molecular analysis. Nevertheless, the limited number of specimens and losses incurred during mounting, particularly for *Hanseniella* sp. 5 and *Symphylella* sp. 2, precluded their inclusion in the molecular analysis.

We reviewed and compared descriptions of known species of the genera *Hanseniella* and *Symphylella* with the collected material, with the objective of raising the identification to the species level when possible. Descriptions and terminology follow Scheller’s descriptions (e.g., Scheller 1961, 1971, 2007); for some structures, terminology follows Snodgrass (1935), Domínguez (2009) and Giurginca (2025).

### Molecular Identification

#### DNA Extraction and PCR amplification

Genomic DNA was extracted from individual symphylan specimens using the DNeasy Blood and Tissue Kit (QIAGEN®, USA), following the manufacturer’s protocol. A ~ 700 base-pair fragment of the mitochondrial cytochrome c oxidase subunit I (COI) gene was amplified via polymerase chain reaction (PCR) using the primers LCO1490 (5’-GGTCAACAAATCATAAAGATATTGG-3’) and HCO2198 (5’-TAAACTTCAGGGTGACCAAAAAATCA-3’) (Folmer et al. 1994) and following the protocol of Ruiz et al. (2010). Each 15 µL PCR reaction contained 1× buffer, 0.5 mM each dNTP, 2 mM MgCl_2_, 0.2 U of Taq polymerase (Invitrogen), 0.3 µM each primer, and 1 µL of DNA template. The PCR thermocycler parameters included an initial denaturation at 95 °C for 5 min, followed by 34 cycles of 95 °C for 30 s, 50 °C for 30 s, and 72 °C for 45 s, and a final extension at 72 °C for 5 min. Amplification success was confirmed by visualizing PCR products on 1.2% agarose gels stained with GelRed (Biotium) using a UV transilluminator.

#### Sequencing and phylogenetic analysis

PCR products exhibiting clear, single bands of the expected size were purified and sent for Bidirectional Sanger sequencing to Corporación Corpogen S.A. (Bogotá, Colombia), using the same primers as in the amplification step. Raw sequence chromatograms were visually inspected, and forward and reverse reads were assembled into consensus sequences using Sequencher® v. 5.1 (Gene Codes Corporation, Ann Arbor, MI, USA). To infer phylogenetic relationships, the generated COI sequences were combined with publicly available symphylan sequences from GenBank, including those from relevant studies in Colombia (Salazar-Moncada et al. 2015) and China (Jin et al. 2023) (Table 5). The outgroup was comprised by several chilopod sequences from the genera *Allothereua*, *Craterostigmus*, *Ethmostigmus*, *Mecistocephalus*, *Pachymerium* and *Lithobius* (Table 5). The final alignment was constructed using MAFFT v. 7 with default settings (Katoh and Standley 2013). Identical sequences were collapsed into unique haplotypes prior to phylogenetic inference.

Phylogenetic inference was performed using the maximum likelihood method in the IQ-TREE web server (http://iqtree.cibiv.univie.ac.at/) with default parameters (Trifinopoulos et al. 2016). The best-fit model of nucleotide substitution, GTR+F+I+G4, was identified using ModelFinder based on the Bayesian Information Criterion (BIC) (Schwarz 1978; Kalyaanamoorthy et al. 2017). The final tree was rooted using the chilopod *Lithobius forficatus* GenBank accession number KM611799.1. Branch support was assessed with the ultrafast bootstrap approximation (UFBoot2) using 1,000 replicates (Hoang et al. 2018). The resulting tree was visualized and annotated in R v. 4.3.1 (R Core Team 2023) using the packages “ape” (Paradis and Schliep 2019), “ggtree” (Yu et al. 2017), “ggplot2” (Wickham 2016), “cowplot” (Wilke 2020), and “treeio” (Wang et al. 2020). The final figure was prepared for publication in Adobe Illustrator (Adobe Inc.).

#### Genetic distance analysis

To quantify the genetic divergence among the identified lineages and assess the presence of a “barcoding gap,” pairwise genetic distances were calculated using the Kimura 2-parameter (K2P) model (Kimura 1980) with the ‘ape’ package in R (Paradis and Schliep 2019). The distribution of intra-clade and inter-clade distances were analyzed by comparing the maximum pairwise distance within each of the major monophyletic clades (recovered in the phylogenetic analysis) to the minimum pairwise distance between individuals of different clades. To visualize these patterns of genetic divergence, a heatmap was generated using the ‘pheatmap’ package (Kolde 2019), with sequences ordered to match the topology of the phylogenetic tree.

### Species descriptions

For the description of the species, photographs of the slides were taken using an Axiocam ERc 5s camera on a Zeiss Primo Star microscope, and measurements were made using Zeiss Zen software v. 3.8. Illustrations were created using Adobe Illustrator. Measurements and ratios given here are those of the holotype, followed by the range of other specimens in the type series in parentheses.

## Results

### Morphological Identification

A total of 992 symphylan specimens were collected from the four pineapple-producing localities in Santander. Morphological examination revealed the presence of two genera and eight distinct morphospecies within the collected samples (Table 2). The locality of Girón (Chocoita) exhibited the highest symphylan diversity, with six morphospecies identified, followed by the two localities of Lebrija, both with three morphospecies. In contrast, only a single morphospecies was found in Villanueva (Table 2). The most abundant morphospecies was *Hanseniella* sp. 1, which was found in all four localities and comprised 95.9% of the adults. While, in contrast, *Symphylella* morphospecies were observed in lower numbers overall, and only across Girón and Lebrija localities.

**Table 2.** Symphylans morphospecies identified in the pineapple-producing area of the department of Santander, Colombia.

Morphospecies	Girón, Chocoita	Lebrija, La Aguada	Lebrija, La Puente	Villanueva	Total	% Adults Abundance
*Hanseniella* sp. 1	13	275	450	17	755	95.9
*Hanseniella* sp. 2	1	4	8	–	13	1.65
*Hanseniella* sp. 3	3	–	–	–	3	0.38
*Hanseniella* sp. 4	4	–	–	–	4	0.51
*Hanseniella* sp. 5	4	–	–	–	4	0.51
*Symphylella* sp. 1	–	2	–	–	2	0.25
*Symphylella* sp. 2	3	–	–	–	3	0.38
*Symphylella* sp. 3	–	–	3	–	3	0.38
Inmatures	15	70	113	8	206	–
**TOTAL**	35	350	578	25	992	–

### Morphological differentiation of *Hanseniella* species

As anticipated, species-level separation and identification within the genus *Hanseniella* proved challenging due to the high degree of morphological similarity among species (Hansen 1903). Accurate differentiation of the five *Hanseniella* morphospecies observed in this study was only possible through careful examination of slide-mounted specimens, with particular attention to antennal chaetotaxy.

**Figure 2.**
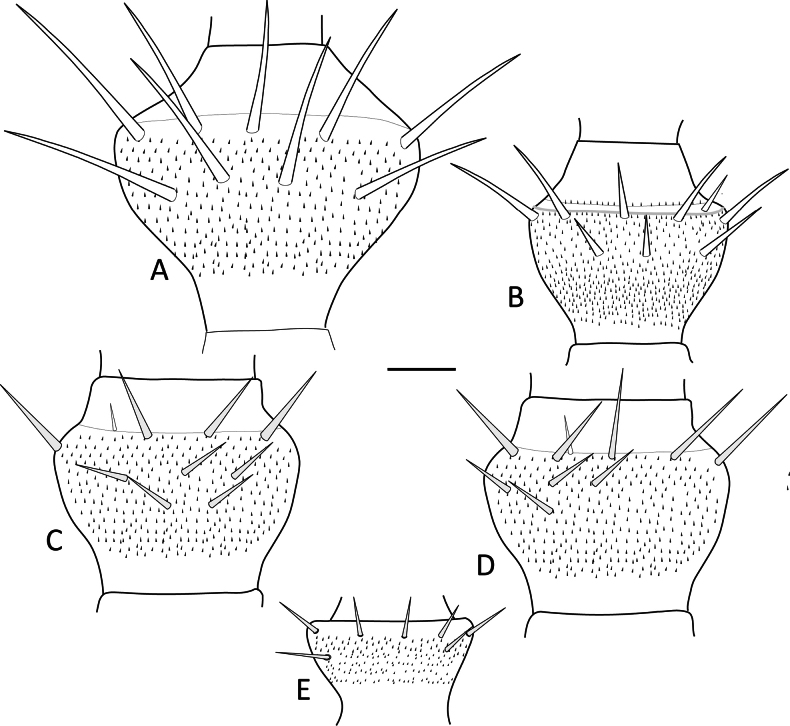
Intermediate antennal segments, ventral view. **A**. *Hanseniella* sp. 1; **B**. *Hanseniella* sp. 2; **C**. *Hanseniella* sp. 3; **D**. *Hanseniella* sp. 4; **E**. *Hanseniella* sp. 5. Scale bar: 20 µm.

**Figure 3.**
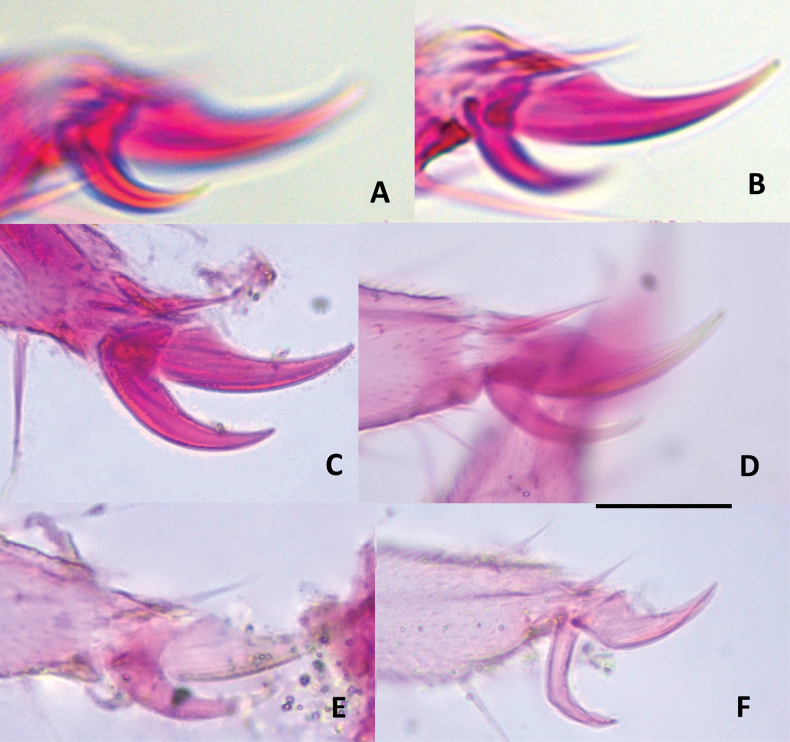
Tarsal claws of 12^th^ pairs of legs of *Hanseniella* morphospecies, anterior view. **A, B**. *Hanseniella* sp. 1; **A**. Posterior claw; **B**. Anterior claw; **C**. *Hanseniella* sp. 2; **D**. *Hanseniella* sp. 3; **E**. *Hanseniella* sp. 4; **F**. *Hanseniella* sp. 5. Scale bar: 20 µm.

Of the 15 morphological characters initially evaluated, only five were ultimately deemed diagnostic for distinguishing among the *Hanseniella* morphospecies. These key characters included: (1) the number of setae on the first tergite, (2) the presence or absence of a third whorl of setae on the intermediate antennal segments (Fig. 2), (3) the length of setae on the secondary whorl of the antennae in relation with the central or primary whorl (Fig. 2) (4) the relative size of the claws on the last pair of legs (Fig. 3), and (5) third scutum emargination (Table 3).

**Table 3.** Morphological characterization of Hanseniella on pinneapple crops in Santander, Colombia.

Morphological character	Reference	* Hanseniella *				
		sp. 1 (*n* = 20)	sp. 2 (*n* = 7)	sp. 3 (*n* = 4)	sp. 4 (*n =* 5)	sp. 5 (*n* = 1)
First tergite setae	Scheller 1961	4	2	5	4	4
Third whorl of setae on intermediate antennal segments	Hansen 1903; Scheller 1961	Absent	Absent	Present	Present	Absent
Setae length of antennae secondary whorl vs central whorl	Hansen 1903	Large	Short	Short	Short	Large
Difference between claws size of the last pair of legs	Hansen 1903	Different	Similar	Different	Different	Different
Head shape	Soesbergen 2019	Heart-shaped	Heart-shaped	Heart-shaped	Heart-shaped	Heart-shaped
Central rod of head	Scheller 2007; Soesbergen 2019	Present	Present	Present	Present	Present
Macrochaetae on tibia and femur	Adam and Burtel 1956	Absent	Absent	Absent	Absent	Present
Setae shape on the dorsal head	Soesbergen 2019	Pointed	Pointed	Pointed	Pointed	Pointed
Styli setae	Soesbergen 2019	2	2	2	2	2
Lateral macrochaetae only on tergites 2, 3, 4, 6, 7, 9	Scheller 1961	Present	Present	Present	Present	Present
Cerci tip	Hansen 1903	Glabrous, conical	Glabrous, conical	Glabrous, conical	Glabrous, conical	Glabrous conical
Third scutum emargination	Scheller 1961	Absent	Absent	Absent	Absent	Present
Penultimate tergite emargination	Scheller 1961	Present	Present	Present	Present	Present
Apical setae length of cerci / Cerci base length. Hansen (2003) has two options: (i) the apical setae of the cerci is at least two-thirds the width of the cerci (ii) or less than half.	Hansen 1903; Soesbergen 2019	0.76–0.86	0.59–0.74	0.62–0.85	0.63–0.76	1.04
Cerci length/ Cerci base length ratio	Hansen 1903; Soesbergen 2019	3.1–4.0	2.4–3.2	2.1–3.9	2.7–3.0	3.9

*n* = number of individuals examined.

### Morphological differentiation of *Symphylella* species

**Figure 4.**
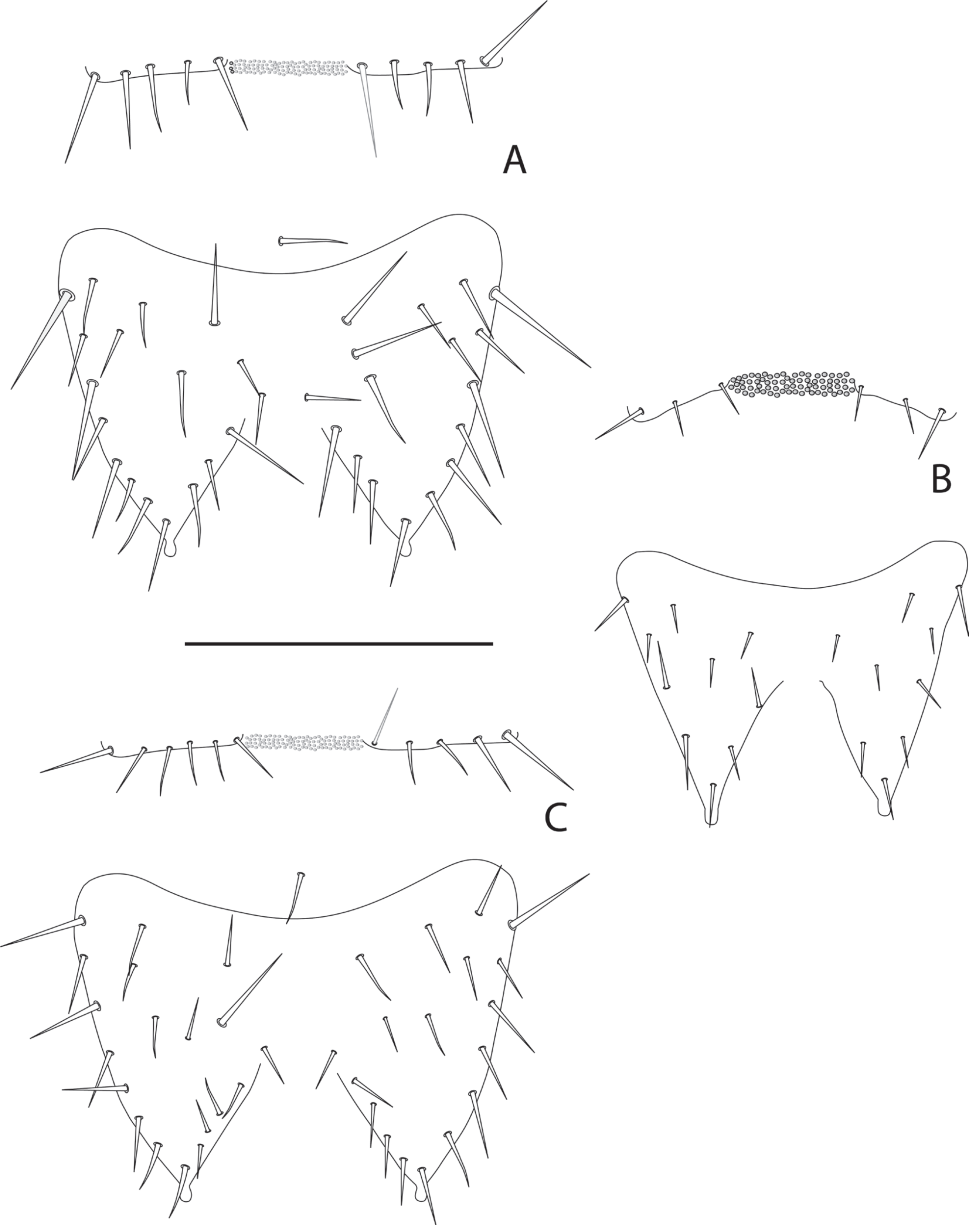
First two tergites and their chaetotaxy in morphospecies of *Symphylella* in Santander, Colombia, dorsal view. **A**. *Symphylella* sp. 1; **B**. *Symphylella* sp. 2; **C**. *Symphylella* sp. 3. Scale bar: 100 µm.

In contrast to *Hanseniella*, the identification of *Symphylella* species relied primarily on the chaetotaxy of the first and second tergites. In total, three distinct *Symphylella* morphospecies were identified (Table 4). *Symphylella* sp. 2 was clearly distinct from the other morphospecies, characterized by a lower number and shorter length of setae on tergites, legs, and cerci. In contrast, *Symphylella* sp. 1 and *Symphylella* sp. 3 exhibited a high degree of morphological similarity, yet they could be differentiated by subtle but consistent differences in the number of setae on the first tergite and on the inner border between the basal and apical setae on second tergite (Table 4, Fig. 4).

**Table 4.** Morphological characterization of *Symphylella* collected from pineapple crops in Santander, Colombia.

Morphological character	Reference	*Symphylella* sp. 1 (*n* = 2)	*Symphylella* sp. 2 (*n* = 2)	*Symphylella* sp. 3 (*n* = 2)
Central rod of head	Scheller 1961	Present	Present	Present
Length of first vs second antennal segment	Scheller 1961	First smaller	First smaller	First smaller
Inner long setae on first antennal segments	Scheller 1986	Bigger than exterior setae	Bigger than exterior setae	Bigger than exterior setae
First tergite setae	Edwards 1959; Domínguez and Vandenspiegel 2012	10	6	11
Posterior tip shape of the triangular tergal processes	Scheller, 1961	Extended, rounded	Extended, rounded	Extended, rounded
Posterior setae between inner basal and apical setae on second tergite	Edwards 1959; Scheller 1971; Domínguez and Vandenspiegel 2012	2–3	1	3
Lateromarginal setae on second tergite	Domínguez and Vandenspiegel 2012	6–7	4	6-7
Second tergite posterior margin shape	Domínguez and Vandenspiegel 2012	Curved	Curved	Curved
Anterolateral setae length on second tergite	Modified from Scheller 1961. Scheller compares them with the length of the triangular process.	Bigger than lateromarginals	Same length as lateromarginals	Bigger than lateromarginals
Presence of a secondary whorl on antennae	Hansen 1903	No secondary whorl	No secondary whorl	No secondary whorl
Setae number on external margin of last tarsus. Edwards 1959, have two options: (i) with four setae, (ii) fewer than four setae.	Edwards 1959	6	5	6
Styli shape	Domínguez and Vandenspiegel 2012	Cylindrical, pointed	Conical, pointed	Cylindrical, pointed
Shape and size difference between claws of the last pair of legs	Scheller 1961	Similar shape, different size	Similar shape and size	Similar shape, different size
Setae number on cerci	Scheller 1961	103–107	44–56	79–87

*n* = number of individuals examined.

### Molecular identification and phylogenetic analysis

A fragment of the mitochondrial cytochrome c oxidase subunit I (COI) gene was sequenced from 13 specimens representing six of eight morphospecies identified in this study. These sequences, along with 35 publicly available sequences from GenBank (Table 5), were aligned using MAFFT to construct a phylogenetic tree.

**Figure 5.**
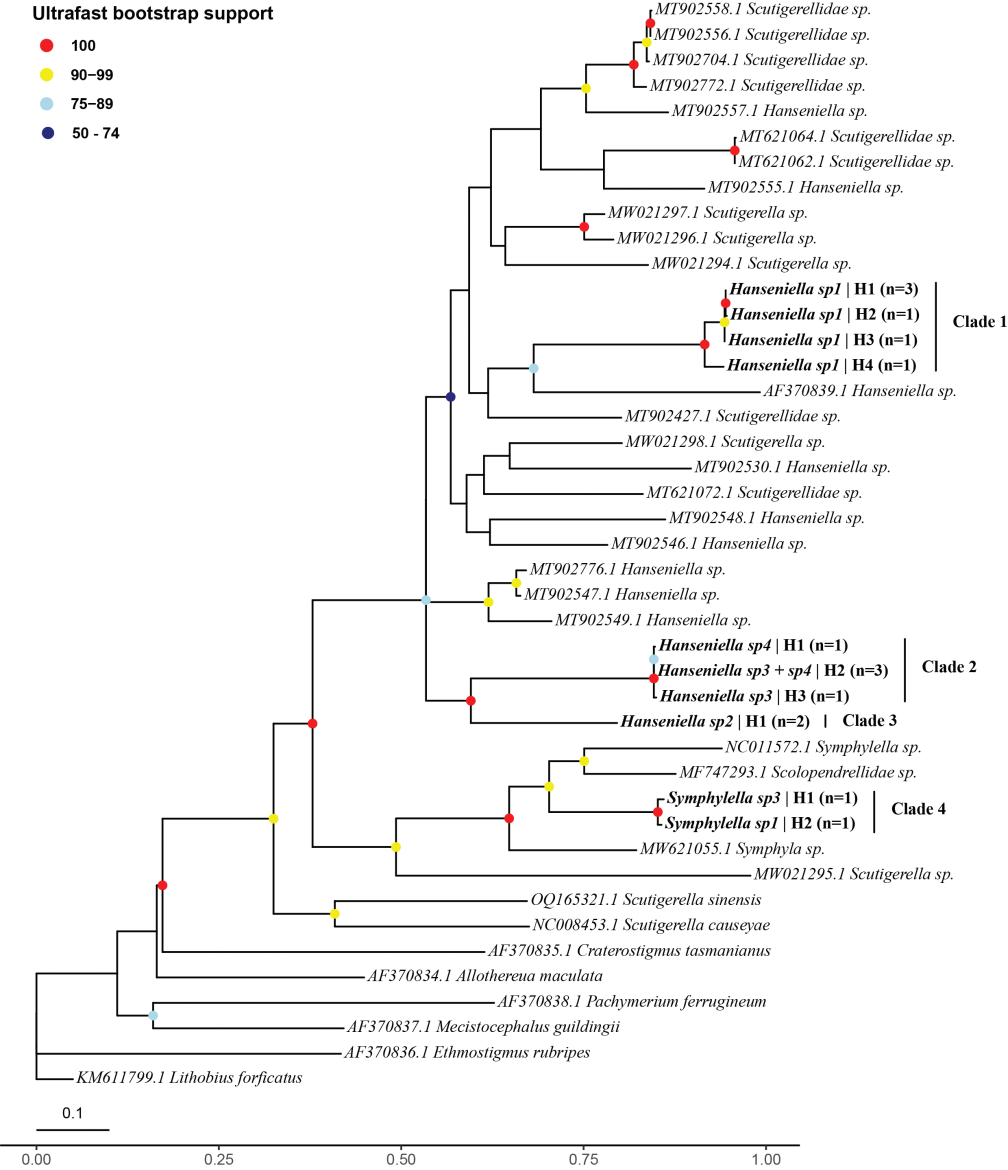
Maximum likelihood phylogenetic tree of symphylans based on an alignment of partial COI gene sequences. Support values at the nodes, indicated by colored circles, represent ultrafast bootstrap percentages from 1,000 replicates. The four major clades containing specimens from this study are indicated on the right. Tip labels for sequences generated in this study indicate the morphospecies designation, a unique haplotype ID (e.g., C1-H1), and the number of individuals (n) sharing that haplotype. GenBank accession numbers are provided for previously published sequences. A full list of specimen identifiers corresponding to each haplotype is provided in Table 5. The scale bar represents the number of nucleotide substitutions per site.

The phylogenetic analysis was based on an alignment of 48 sequences and 667 base pairs. The maximum likelihood analysis recovered the class Symphyla as a strongly supported monophyletic group, distinct from the class Chilopoda represented by sequences of the genera *Lithobius*, *Allothereua*, *Craterostigmus*, *Ethmostigmus*, *Mecistocephalus*, and *Pachymerium* (Fig. 5). The symphylan sequences from this study clustered into four distinct clades. Clade 1 (100% support) comprised all individuals of *Hanseniella* sp. 1. This clade showed distinct haplotypes (H1–H4). The most common haplotype (H1) was shared by three individuals from this study and two previously published sequences from the Colombian Andes (Salazar-Moncada et al. 2015), while the other three haplotypes (H2, H3, and H4) were unique to single individuals. Clade 2 (100% support) contained individuals assigned to morphospecies *Hanseniella* sp. 3 and *Hanseniella* sp. 4. This clade also exhibited genetic diversity, resolving into three distinct haplotypes (H1–H3). The most common of these (H2) was shared by three individuals belonging to both morphospecies (*Hanseniella* sp. 3 and sp. 4). The other two haplotypes were unique to single individuals identified as *Hanseniella* sp. 4 (H1) and *Hanseniella* sp. 3 (H3). Clade 3 (99% support), the sister group to Clade 2, was composed of individuals identified as *Hanseniella* sp. 2. The two sequenced specimens from this morphospecies were genetically identical, sharing a single haplotype (H1), and Clade 4 (100% support) was recovered within the Scolopendrellidae and included the two sequenced *Symphylella* specimens from this study. The analysis resolved these individuals, identified as *Symphylella* sp. 1 and *Symphylella* sp. 3, as two distinct sister haplotypes (H2 and H1, respectively). This clade was sister to a group containing other Scolopendrellidae from GenBank, including a *Symphylella* from China and an unidentified specimen from Canada.

### Genetic distance and heatmap visualization

**Figure 6.**
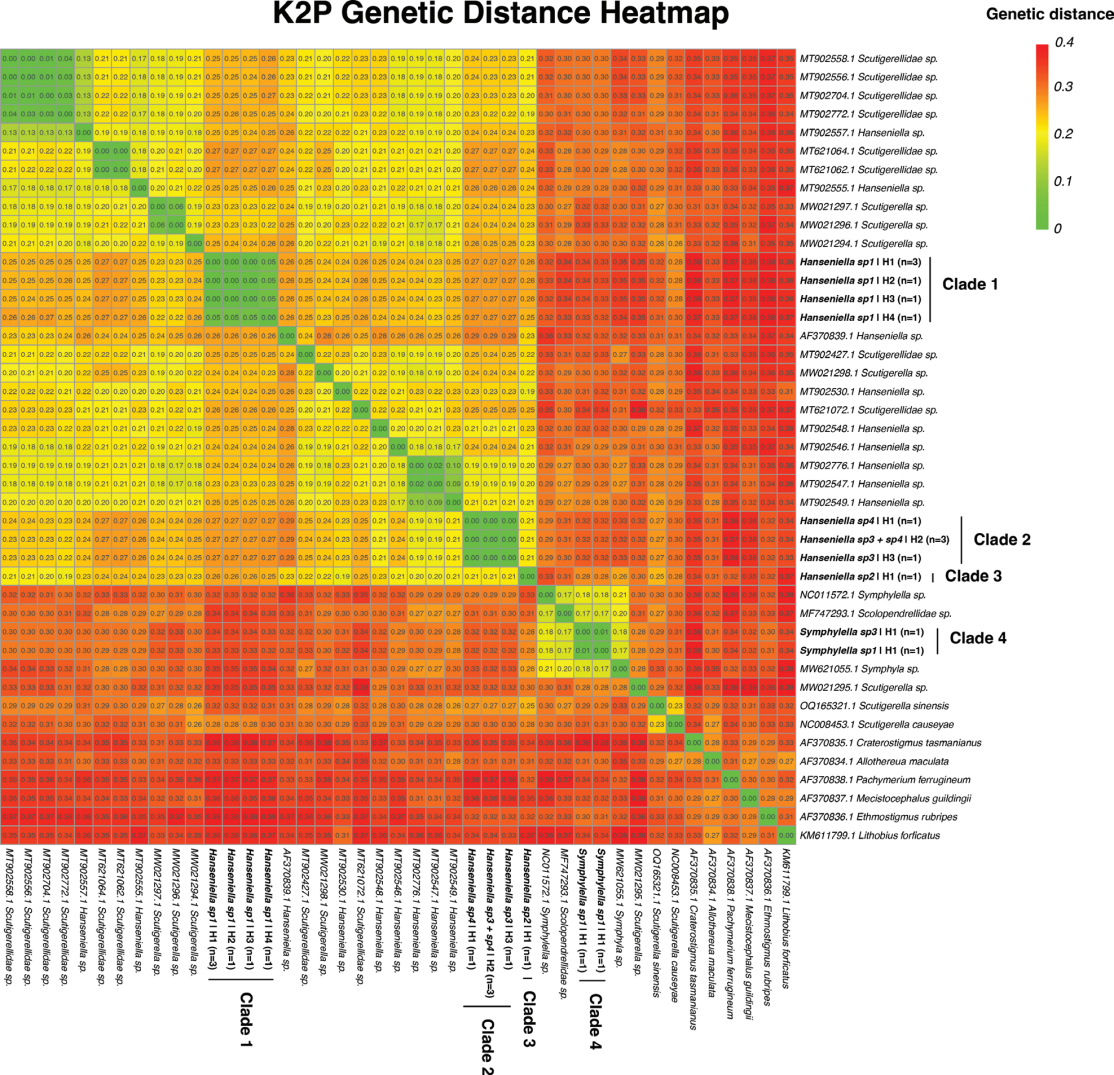
Heatmap visualizing pairwise Kimura 2-parameter (K2P) genetic distances between COI sequences of symphylans and outgroups. Red colours indicates a higher genetic distance, yellow intermediate and green low distances.The arrangement of sequences is based on the topology of the phylogenetic tree (Fig. 5), highlighting the low intra-clade and high inter-clade divergence among the four focal clades.

Pairwise Kimura 2-parameter (K2P) genetic distances were calculated to quantify the genetic divergence among the identified lineages (Fig. 6). The analysis revealed a clear separation between intra-clade and inter-clade divergence values. Intra-clade K2P distances were low. The maximum divergence within Clade 1 (*Hanseniella* sp. 1) was 1.0%, and within Clade 2 (*Hanseniella* sp. 3 + sp. 4), it was also 1.0%. The distance between the two haplotypes in Clade 4 (*Symphylella*) was 1.0%. Clade 3 (*Hanseniella* sp. 2) consisted of a single haplotype with 0% internal divergence. In contrast, inter-clade distances were substantially higher. The minimum K2P distance between Clade 1 and its sister group (Clades 2 + 3) was 17%. The minimum distance between the sister lineages, Clade 2 and Clade 3, was 17%. The minimum divergence between the *Hanseniella* clades (Clades 1, 2, and 3) and the *Symphylella* clade (Clade 4) was 25%. The genetic distance between all symphylan taxa and the chilopod outgroup was even greater, ranging from 25% to 39%, confirming a deep evolutionary divergence between the two classes.

### Congruence between morphological and molecular data

An integrative approach was used to delimit the final species boundaries by reconciling the initial morphological assessments with the molecular data. While morphological examination initially identified eight distinct morphospecies, the phylogenetic analysis and genetic distance data clarified the status of several of these groups. The molecular data did not support the separation of *Hanseniella* sp. 3 and *Hanseniella* sp. 4, which were recovered as a single, genetically cohesive lineage (Clade 2). Similarly, *Symphylella* sp. 1 and *Symphylella* sp. 3 were resolved as distinct but closely related sister haplotypes within a single lineage (Clade 4). Based on this evidence, the minor morphological differences between these pairs are considered to represent intraspecific variation. Consequently, from the initial eight morphospecies, six were successfully sequenced and correspond to four well-supported genetic clades identified in the phylogenetic analysis. The complete synthesis of these findings, including key genetic distance metrics, is summarized in Table 6.

**Table 5.** Symphylan and outgroup sequences included in the final phylogenetic analysis, with corresponding haplotype assignments for newly sequenced specimens. Haplotype IDs are designated by clade number (C1–C4) and haplotype number within that clade (H1, H2, etc.) as shown in Figure 5.

Haplotype ID	Species and voucher	Family	Genus	Country	GenBank number	CTNI specimen number
C1-H1	*Hanseniella* sp. 1 (Chocoita 24)	Scutigerellidae	* Hanseniella *	Colombia	PV612820	10410a
C1-H2	*Hanseniella* sp. 1 (La puente 40)	Scutigerellidae	* Hanseniella *	Colombia	PV612814	10410b
C1-H3	*Hanseniella* sp. 1 (La puente 4)	Scutigerellidae	* Hanseniella *	Colombia	PV612813	10410c
C1-H4	*Hanseniella* sp. 1 (Chocoita 30)	Scutigerellidae	* Hanseniella *	Colombia	PV612815	10410d
C2-H1	*Hanseniella* sp. 4 (Chocoita 20)	Scutigerellidae	* Hanseniella *	Colombia	PV612822	10413b
C2-H2	*Hanseniella* sp. 3 (Chocoita 27)	Scutigerellidae	* Hanseniella *	Colombia	PV612816	10412a
–	*Hanseniella* sp. 3 (Chocoita 26)	Scutigerellidae	* Hanseniella *	Colombia	PV612817	10412b
–	*Hanseniella* sp. 4 (Chocoita 21)	Scutigerellidae	* Hanseniella *	Colombia	PV612821	10413a
C2-H3	*Hanseniella* sp. 3 (Chocoita 19)	Scutigerellidae	* Hanseniella *	Colombia	PV612818	10412c
C3-H1	*Hanseniella* sp. 2 (La puente 10)	Scutigerellidae	* Hanseniella *	Colombia	PV612810	10411a
–	*Hanseniella* sp. 2 (Chocoita 32)	Scutigerellidae	* Hanseniella *	Colombia	PV612819	10411b
C4-H1	*Symphylella* sp. 3 (La puente 45)	Scolopendrellidae	* Symphylella *	Colombia	PV612812	10415
C4-H2	*Symphylella* sp. 1 (La puente 44)	Scolopendrellidae	* Symphylella *	Colombia	PV612811	10414
–	*Hanseniella* sp.	Scutigerellidae	* Hanseniella *	Unknown	AF370839.1	–
–	Scutigerellidae sp. (FRL–2015)	Scutigerellidae	Undetermined	Colombia	KP696390.1	–
–	Scutigerellidae sp.	Scutigerellidae	Undetermined	Colombia	KP696391.1	–
–	Scolopendrellidae sp.	Scolopendrellidae	Undetermined	Canada	MF747293.1	–
–	Symphyla sp. (DNA07)	Symphyla	Undetermined	Australia	MW621055.1	–
–	Scutigerellidae sp. (BMR01576)	Scutigerellidae	Undetermined	Australia	MT621062.1	–
–	Scutigerellidae sp. (BMR01578)	Scutigerellidae	Undetermined	Australia	MT621064.1	–
–	Scutigerellidae sp. (BMR01587)	Scutigerellidae	Undetermined	Australia	MT621072.1	–
–	Scutigerellidae sp. (BMR00071)	Scutigerellidae	Undetermined	Australia	MT902427.1	–
–	*Hanseniella* sp. (BMR00202)	Scutigerellidae	* Hanseniella *	Australia	MT902530.1	–
–	*Hanseniella* sp. (BMR00229)	Scutigerellidae	* Hanseniella *	Australia	MT902546.1	–
–	*Hanseniella* sp. (BMR00230)	Scutigerellidae	* Hanseniella *	Australia	MT902547.1	–
–	*Hanseniella* sp. (BMR00231)	Scutigerellidae	* Hanseniella *	Australia	MT902548.1	–
–	*Hanseniella* sp. (BMR00232)	Scutigerellidae	* Hanseniella *	Australia	MT902549.1	–
–	*Hanseniella* sp. (BMR00241)	Scutigerellidae	* Hanseniella *	Australia	MT902555.1	–
–	Scutigerellidae sp. (BMR00242)	Scutigerellidae	Undetermined	Australia	MT902556.1	–
–	*Hanseniella* sp. (BMR00243)	Scutigerellidae	* Hanseniella *	Australia	MT902557.1	–
–	Scutigerellidae sp. (BMR00244)	Scutigerellidae	Undetermined	Australia	MT902558.1	–
–	Scutigerellidae sp. (BMR00641)	Scutigerellidae	Undetermined	Australia	MT902704.1	–
–	Scutigerellidae sp. (BMR01199)	Scutigerellidae	Undetermined	Australia	MT902772.1	–
–	*Hanseniella* sp. (BMR01208)	Scutigerellidae	* Hanseniella *	Australia	MT902776.1	–
–	*Scutigerella* sp. (WAMT144261)	Scutigerellidae	* Scutigerella *	Australia	MW021294.1	–
–	*Scutigerella* sp. (WAMT144298)	Scutigerellidae	* Scutigerella *	Australia	MW021295.1	–
–	*Scutigerella* sp. (WAMT145461)	Scutigerellidae	* Scutigerella *	Australia	MW021296.1	–
–	*Scutigerella* sp. (WAMT145462)	Scutigerellidae	* Scutigerella *	Australia	MW021297.1	–
–	*Scutigerella* sp. (WAMT145463)	Scutigerellidae	* Scutigerella *	Australia	MW021298.1	–
–	*Scutigerella causeyae*	Scutigerellidae	* Scutigerella *	Germany	NC_008453.1	–
–	*Symphylella* sp. (YG–2006)	Scolopendrellidae	* Symphylella *	China	NC_011572.1	–
–	*Scutigerella sinensis*	Scutigerellidae	* Scutigerella *	China	OQ165321.1	–
–	*Allothereua maculata*	Scutigeridae	* Allothereua *	Unknown	AF370834.1	–
–	*Craterostigmus tasmanianus*	Craterostigmidae	* Craterostigmus *	Unknown	AF370835.1	–
–	*Ethmostigmus rubripes*	Scolopendridae	* Ethmostigmus *	Unknown	AF370836.1	–
–	*Mecistocephalus guildingii*	Mecistocephalidae	* Mecistocephalus *	Unknown	AF370837.1	–
–	*Pachymerium ferrugineum*	Geophilidae	* Pachymerium *	Unknown	AF370838.1	–
–	*Lithobius forficatus*	Lithobiidae	* Lithobius *	Canada	KM611799.1	–

CTNI= Colección Taxonómica Nacional de Insectos “Luis Maria Murillo”.

**Table 6.** Integrative taxonomic synthesis of symphylans from pineapple crops in Santander, Colombia.

Initial morphospecies	Molecular lineage (clade)	Key Genetic Distances (K2P)	Final Taxonomic Assignment
Genus *Hanseniella*			
*Hanseniella* sp. 1	Clade 1	Max. intra-clade: 1.0%	*Hanseniella cf. unguiculata* Hansen, 1903
*Hanseniella* sp. 2	Clade 3	Intra-clade: 0%	*Hanseniella lebrijana* sp. nov.
*Hanseniella* sp. 3 & sp. 4	Clade 2	Max. intra-clade: 1.0%	*Hanseniella chocoita* sp. nov.
*Hanseniella* sp. 5	Not sequenced	Not applicable	*Hanseniella* sp. 5
Genus *Symphylella*			
*Symphylella* sp. 1 & sp. 3	Clade 4	Intra-clade: 1.0%	*Symphylella* sp. 1
*Symphylella* sp. 2	Not sequenced	Not applicable	*Symphylella* sp. 2

### Taxonomy

#### Subphylum Myriapoda Latreille, 1797

**Class Symphyla Ryder, 1880**

**Family Scutigerellidae Bagnall, 1913**

**Genus *Hanseniella* Bagnall, 1913**

##### Hanseniella cf. unguiculata

Taxon classificationAnimaliaSymphylaScutigerellidae

Hansen, 1903

DFC07F95-4BDD-52EC-9080-828D12BCFD14

###### Material studied.

• 3 females, 2 males (CTNI 10308 a-e), **Colombia**: Santander, Lebrija, Vereda La Aguada de Ceferino, manual collection in pineapple farming, 975 m a.s.l., 7.1978, -73.1714, 15-X-2019, J. Montes-Rodríguez; • 1 female, 1 male, **Colombia**: Santander, Lebrija, Vereda La Aguada de Ceferino, manual collection in pineapple farming, 975 m a.s.l., 7.1978, -73.1714, 15-X-2019, J. Montes-Rodríguez, LELaAgu01-9 (Field code); • 1 female, 1 male (CTNI 10410 a, d) **Colombia**: Santander, Girón, Vereda Chocoita, manual collection in pineapple farming, 850 m a.s.l., 6.9867, -73.1628, 15-IX-2020, J. Montes-Rodríguez; • 1 female, 1 male, (CTNI 10410 b, c) **Colombia**: Santander, Lebrija, Vereda la Puente, manual collection in pineapple farming, 1297 m a.s.l., 7.0790, -73.2033, 15-XI-2019, J. Montes-Rodríguez.

###### Distribution.

This species was collected in the municipalities of Villanueva, Girón, and Lebrija. It was the dominant species in pineapple crops in the department of Santander. The similarity of their sequences to those published by Salazar-Moncada et al. (2015) suggests that this species has a wide distribution in Colombia.

###### Remarks.

Specimens from Santander clearly exhibit the key diagnostic characters traditionally used to identify this species, namely the shape of the claw on leg 12 and macrochaetal pattern. However, Hansen’s (1903) original description of specimens from Venezuela is rather brief, and the accompanying illustrations are insufficient to confirm the identification with complete certainty. Edwards (1959) subsequently provided more detailed documentation, including measurements of tergites, lateral macrochaetae, and new illustrations, although based on material from England. The morphological ranges reported by Edwards (1959) generally overlap with those observed in the Santander specimens, and the illustrations are reasonably consistent with the new material. Nevertheless, in the absence of a modern redescription of the type specimens, we refrain from definitively assigning the Santander material to *H. unguiculata*. Despite this taxonomic uncertainty, the species appears strongly associated with pineapple cultivation in Santander, where it was the dominant symphylan in sampled crops. Records from Hawaii, where it was the only *Hanseniella* species reported in pineapple cultivation (Scheller 1961), further support a clear ecological relationship with this host plant. Its broad geographic distribution and occurrence across multiple continents have made it one of the most frequently cited symphylan species in the literature; however, its biogeographic origin and native or introduced status in Colombia and other parts of the world remain unresolved.

##### Hanseniella lebrijana sp. nov.

Taxon classificationAnimaliaSymphylaScutigerellidae

CDD0231D-7785-5AC0-A47A-D642B296ED1D

https://zoobank.org/AA915426-8E13-465F-B413-0D5EB7755F45

**Figure 7.**
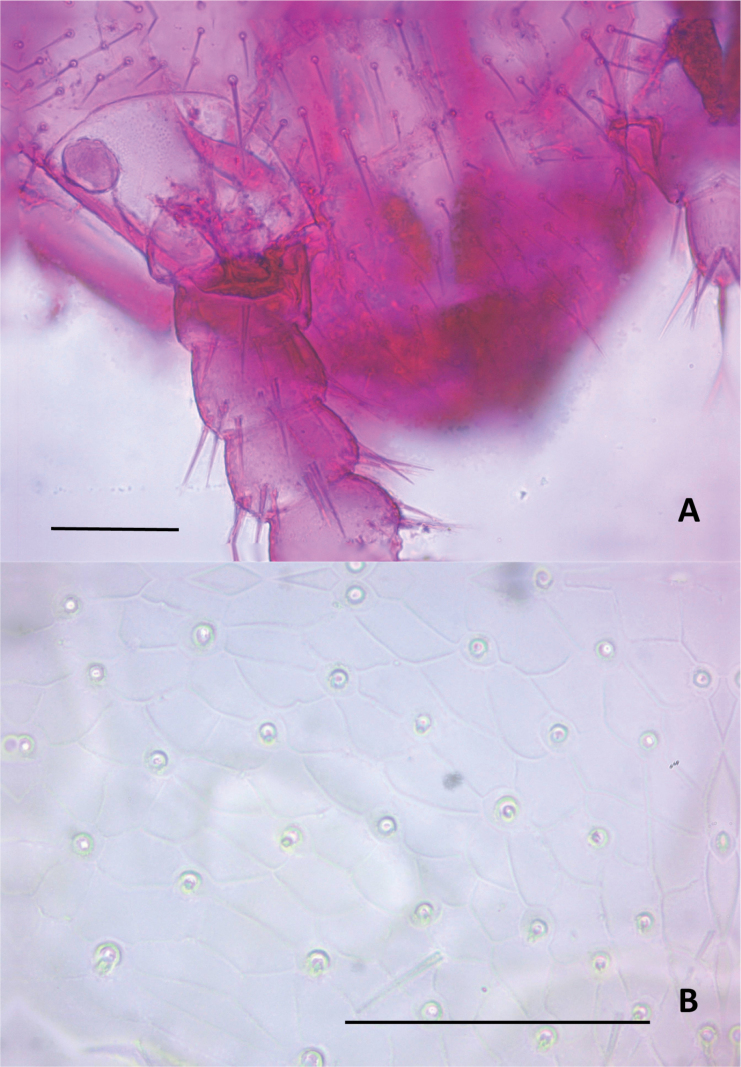
Head of *Hanseniella lebrijana* sp. nov. **A**. Anterior dorsal surface of the head showing the base of the antenna, dorsal view (paratype); **B**. Cuticle on the anterior dorsal part of the head between the antennae, dorsal view (paratype). Scale bars: 50 µm.

**Figure 8.**
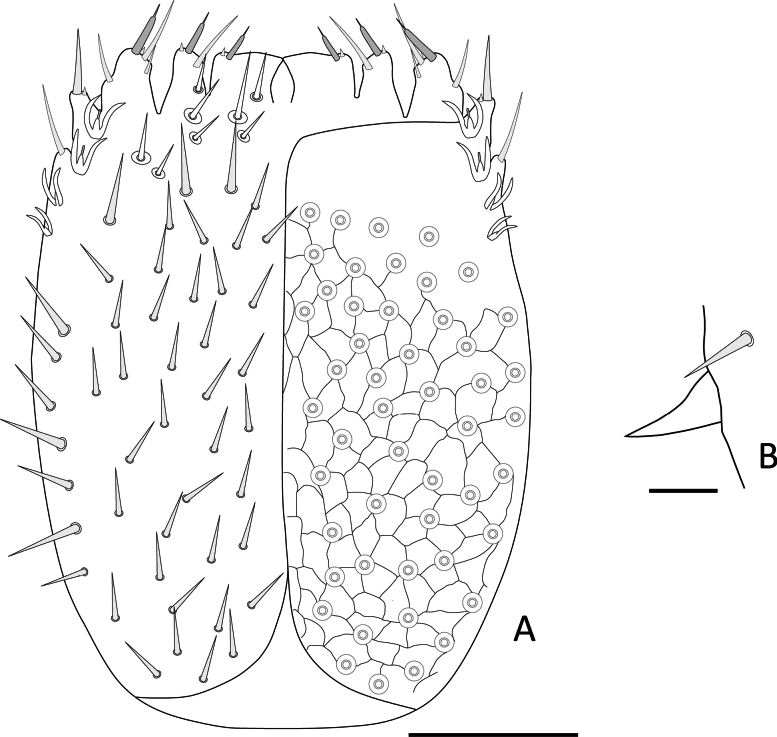
Anterior plates of the second maxillae of *Hanseniella lebrijana* sp. nov. **A**. Surface and setae of the second maxilla. Left half with setae and right half with micro-sculpture of the cuticle, ventral view; **B**. Conical palp of the first maxilla, ventral view. Scale bars: 50 µm (**A**); 10 µm (**B**).

**Figure 9.**
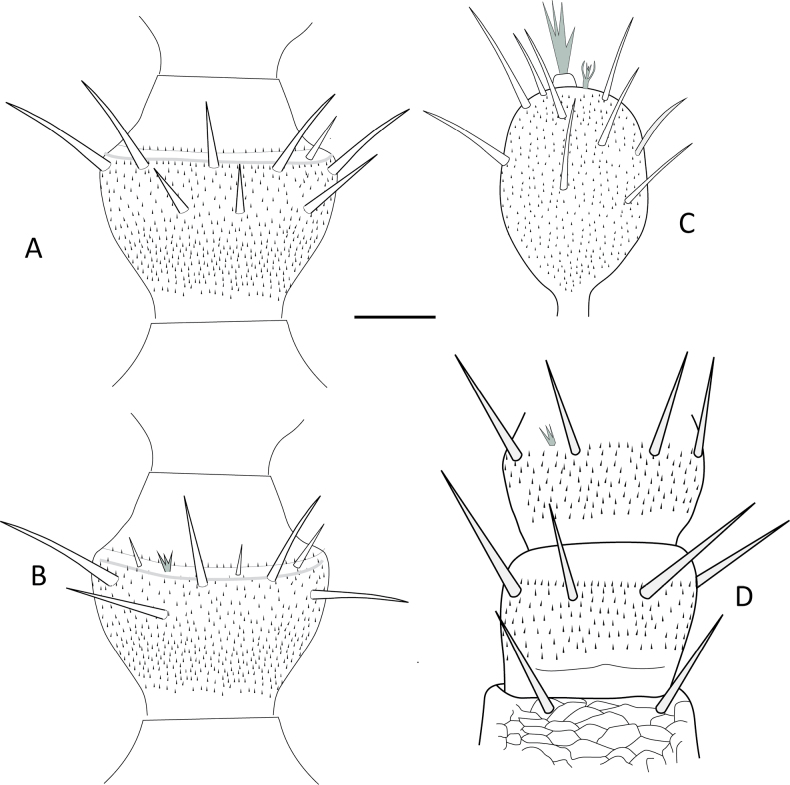
Antennae of *Hanseniella lebrijana* sp. nov. **A, B**. Intermediate segments (ventral and dorsal respectively); **C**. Last segment, dorsal view; **D**. Basal antennal segments, dorsal view. Scale bar: 20 µm

**Figure 10.**
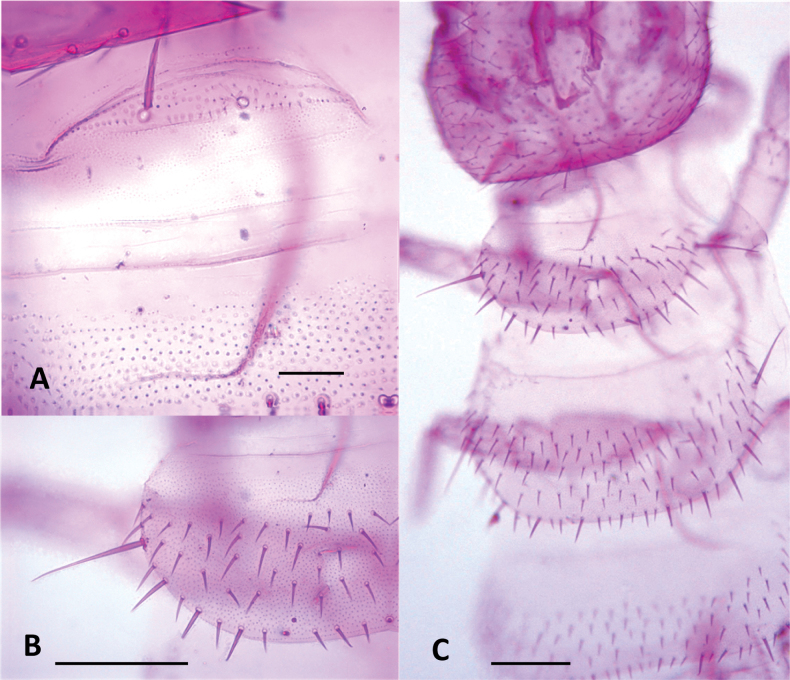
Tergites of *Hanseniella lebrijana* sp. nov. **A**. First rudimentary tergite and part of second tergite, dorsal view (holotype); **B**. Second tergite left half, dorsal view (holotype); **C**. First four tergites, dorsal view (holotype). Scale bars: 20 µm (**A**); 100 µm (**B, C**).

**Figure 11.**
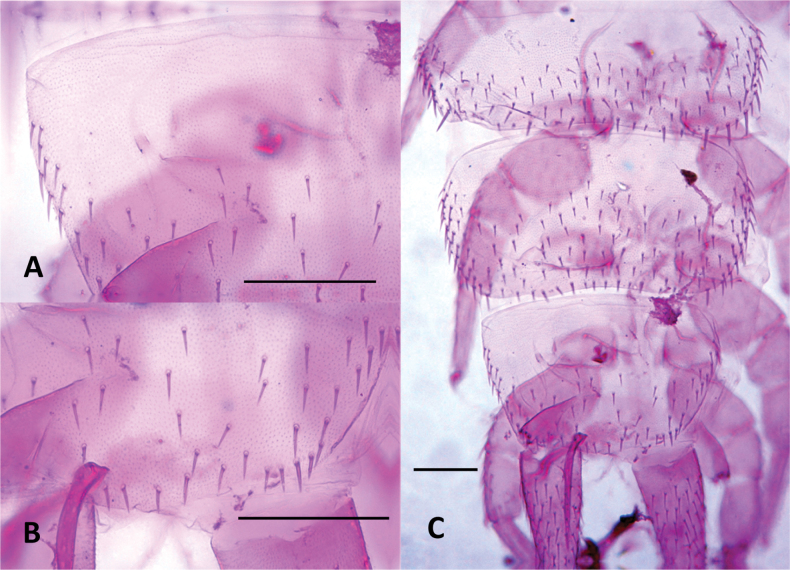
Last tergites of *Hanseniella lebrijana* sp. nov. holotype **A**. Left upper half of the last tergite, dorsal view; **B**. Last tergite near the base of the cerci, dorsal view; **C**. Last tergites, dorsal view. Scale bars: 100 µm.

**Figure 12.**
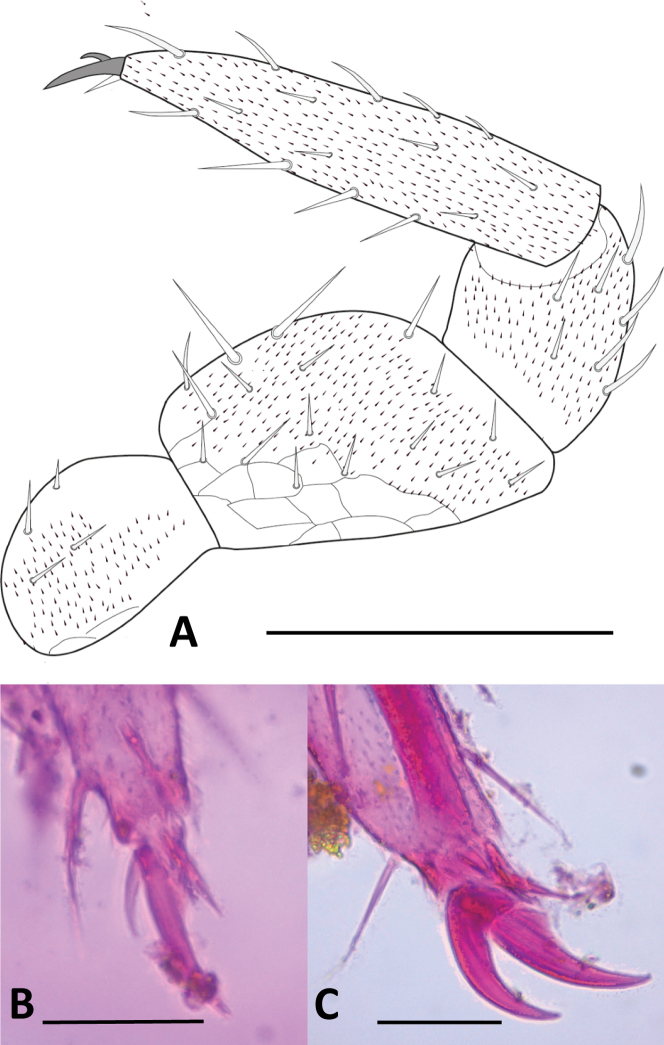
Legs of *Hanseniella lebrijana* sp. nov. **A**. Posterior view of first left leg; **B**. Anterior view of claws on the first pair of legs (holotype); **C**. Anterior view of claws on the last pair of legs (paratype). Scale bars: 100 µm (**A**); 20 µm (**B**); 50 µm (**C**).

**Figure 13.**
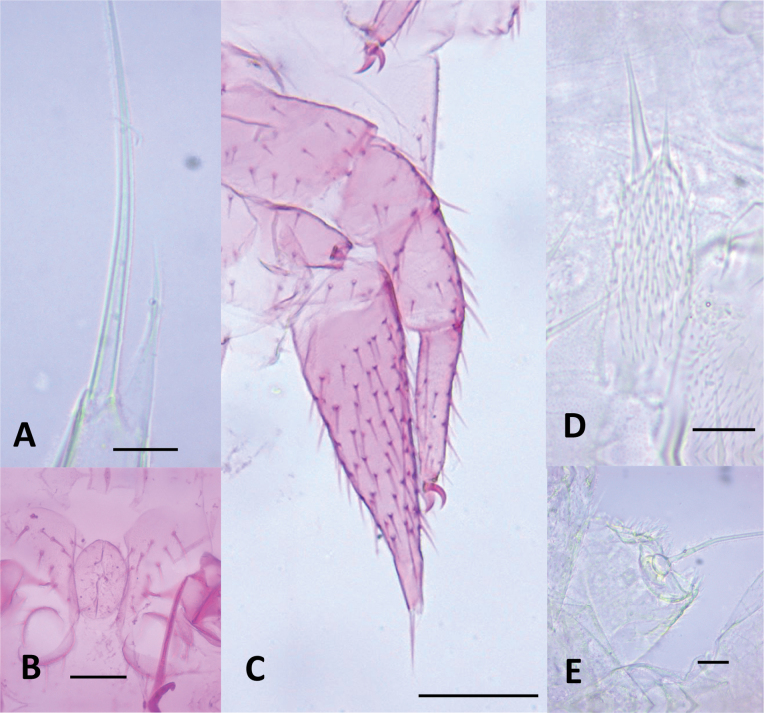
Legs and ventral surface of *Hanseniella lebrijana* sp. nov. paratype. **A**. Dorsal view of apex of the cerci; **B**. Male organ; **C**. Ventral view of cerci and last pair of legs; **D**. Styli; **E**. Dorsal view of sense calicles. Scale bars: 10 µm (**A, D, E**); 50 µm (**B**); 100 µm (**C**).

Figs 7, 8, 9, 10, 11, 12, 13

###### Type material.

***Holotype*** • female (CTNI-10573a), **Colombia**: Santander, Lebrija, Vereda la Puente, manual collection in pineapple farming, 1297 m a.s.l., 7.0790, -73.2033, 15-XI-2019, J. Montes-Rodríguez. ***Paratypes*** • 3 females, 3 males (CTNI-10573 b-g) same data as for holotype; 1 female (CTNI-10411a) **Colombia**: Santander, Lebrija, Vereda la Puente, manual collection in pineapple farming, 1297 m a.s.l., 7.0790, -73.2033, 15-IX-2019, J. Montes-Rodríguez; • 1 male (CTNI-10411a) **Colombia**: Santander, Girón, Vereda Chocoita, manual collection in pineapple farming, 850 m a.s.l., 6.9867, -73.1628, 15-IX-2020. J. Montes-Rodríguez.

###### Type locality.

Lebrija, Santander, Colombia

###### Diagnosis.

*Hanseniella lebrijana* sp. nov. shares the same pattern of macrochaetae on tergites 2–4, 6–7 and 9 with *H. aculeata* Jupeau, 1954, *H. afromontana* Scheller, 1954, *H. armigera* Scheller, 1961, *H. caldaria* (Hansen, 1903), *H. chocoita* sp. nov. Montes, Parra-Gómez, Holguín & Marchant, 2025, *H. colombiana* Juberthie-Jupeau & Réveillet, 1997, *H. conisetosa* Scheller, 1971, *H. echinata* Adam & Burtel, 1956, *H. ghanensis* Belfield, 1988, *H. guimaraensis* Scheller, 2007, *H. hortulana* Scheller, 1971, *H. incompta* Scheller, 1971, *H. ivorensis* Juberthie-Jupeau & Kehe, 1978, *H. lucifuga* Scheller, 1961, *H. milloti* Aubrey & Masson, 1953, *H. modesta* Aubrey & Masson, 1953, *H. montana* Scheller, 1971, *H. orientalis* (Hansen, 1903), *H. remyi* Aubrey & Masson, 1953, *H. similis* Scheller, 1961 and *H. unguiculata* (Hansen, 1903). It differs from them by the following combination of characters: body length 2.9–3.9 mm; distinct central rod on head; first maxillary palp conical and simple; antennae with two whorls maximum, simple setae of varying size and small trifurcate organs; apical antennomere with usual stalked organ, plus a smaller one; first rudimentary tergite with two setae; posterior margin of tergite 2 slightly convex, in tergite 3 mostly straight, in tergites 13 and 14 slightly concave; anterior margin of all tergites glabrous, with micropubescence; sclerites near coxal sacs with simple setae; second podomere of first leg pair with two ventral setae distinctly stronger and larger than the others; claws of 12^th^ pair of legs arched and subequal in overall length, with acuminated tips; styli with two setae, larger seta 2.5× the size of the small one; cerci setose, bearing simple setae and with a significant basal portion glabrous and with micropubescence. It can also be differentiated from the most similar species by: the presence of the central rod in the head, which is absent in *H. guimaraensis*, and in the relative length of the third podomere or femur of the 12^th^ leg, which is very short in *H. guimaraensis*. *H. colombiana*, *H. echinata*, and *H. ghanensis* also shows similarity to *H. lebrijana* sp. nov. but can be easily distinguished by the claws on leg 12, which are noticeably different in size, while they are similar in size in *H. lebrijana* sp. nov. *H. afromontana* differs in size, measuring 4.1–9.6 mm, while *H. lebrijana* measures 2.9–3.9 mm. The cerci in *H. lucifuga* are 3.7× longer than it is wide, whereas in *H. lebrijana* sp. nov. it is 2.4–3.2× longer than wide. *H. caldaria* and *H. orientalis* have three whorls of setae on the intermediate antennomeres, while *H. lebrijana* does not. Finally, in *H. orientalis*, the proximal and distal setae of the cerci are the same length, while in *H. lebrijana* sp. nov. the distal setae are slightly longer.

###### Description.

Length of body without antennae and cerci 3.3 (2.9–3.9) mm, antenna 1.26 (1.26–1.31) mm. ***Head***. Slightly longer than wider, 1.08 (1.06–1.16) × broader than long, frontal margin convex, lateral margin at point of articulation moderately smooth, posterior margin straight with rounded posterolateral angles. Central rod posteriorly ovoid. Dorsal surface smooth without micropubescence or microsculpture, except for the anterior area of the head between the antennae, which has a scale-like cuticle (Fig. 7B). Dorsal surface covered by straight setae not significantly different, few large setae ~ 2.5× longer than normal setae and 0.68× the width of the first antennomer, arranged in 3+3 anteriorly of antennal insertion (Fig. 7A), two near the anterior head margin, and three laterally on each side behind the rounded Tömösváry’s organ (~ 21 microns wide) (Fig. 7A). Each anterior plate of second maxillae with three proximal setae. External-distal corner of these plates with four sets of sensilla with typical chandelier shape decreasing in size proximally and two elongated setae inserted on conic protuberances, the most posterodistal one with a contiguous tiny tooth. Three terminal protuberances with 1–3 setae plus one large distal sensillum, all with a contiguous tiny tooth. First maxillary palp conical (Fig. 8).

***Antennae***. (Fig. 9). Long, 0.26–0.35× the length of the body, composed of 25–32 antennomers. Surface of each antennomere with micropubescence except the first, which has a scale-like cuticle (Fig. 9D). First antennomere ~ 1.8× wider than longer, with a single distal whorl of five setae. Second antennomere ~ 1.4× wider than longer. From second antennomere and on a single whorl of eight thick and long setae, varying between 7–9, absent on last antennomere. Thick setae from the distal whorl accompanied by thinner and shorter setae from segments 2 and 3, initially with one, gradually and slowly increasing in number up to four or five in the penultimate antennomere. Small trifurcate organ present from antennomeres 2 and 3 onwards. Secondary whorl of setae begins at antennomere 5 with two setae and gradually increases to five or six in the intermediate antennomers and only three in the last ones. Apical antennomere spherical, apex bearing a large sensory organ borne from a small protuberance, composed of a central stalk which yields five slightly longer spiniform processes. One additional similar organ smaller and without protuberance (Fig. 9).

***Tergites***. (Figs 10, 11, Table 7) Cuticle with smooth surface, covered in micropubescence. First tergite rudimentary, with only two central setae and very sparse pubescence in a scaly pattern. From second tergite onwards: 0.33–0.5 anterior surface without setae, only with micropubescence or microtrichiae; 0.1 anterior surface of the tergites with microtrichiae in a scaly arrangement and with a well-marked irregular line. Four types of setae of different length present on tergites: (i) small and similar in size, covering the tergal dorsal surface; (ii) ~ 2× as large as the anterior type, sparsely present on the posterior margin; (iii) ~ 1.5× larger than the large marginal ones, located on the posterolateral corners except on tergites 1, 14, and 15; (iv) Macrochaetae in segments 2–4, 6–7, and 9, ~ 2.0× larger than the anterior type. Tergites 6, 9, and 12 longest (less rectangular and squarer) and more setose.

**Table 7.** Description of tergites and number of tergal setae of *Hanseniella lebrijana* sp. nov. Measurements of the holotype and paratypes.

Tergite	Shape	Posterior margin	Total number of setae	Setae in the posterior margin
2	Semicircular	Slightly convex	67 (78–75)	18 (28–24)
3	Semicircular	Almost straight	113 (131–106)	28 (40–32)
4	Subrectangular	Slightly concave	127 (134–99)	40 (43–33)
5	Subrectangular	Slightly concave	87 (93–72)	34 (35–32)
6	Subrectangular	Slightly concave	183 (217–151)	45 (41–40)
7	Subrectangular	Slightly concave	153 (186–138)	43 (47–36)
8	Subrectangular	Slightly concave	99 (102–93)	36 (36–32)
9	Subrectangular	Slightly concave	185 (236–170)	44 (45–42)
10	Subrectangular	Slightly concave	160 (173–134)	42 (45–41)
11	Subrectangular	Slightly concave	90 (108–82)	33 (38–33)
12	Subrectangular	Slightly concave	182 (222–170)	49 (49–48)
13	Subrectangular	Slightly concave	129 (140–116)	43 (42–38)
14	Subrectangular	Slightly concave	134 (159–122)	38 (40–40)
15	Subquadrate	Convex between cerci	64 (100–42)	15 (31–15)

***Legs***. (Figs 12, 13C). First pair of legs composed of four segments. First podomere subrectangular, ~ 1.4× longer than wide, covered with micropubescence and ~ 4 posteroventral setae. Second podomere trapezoidal, ~ 1.3× longer than wide, with scale-like cuticle, surface with micropubescence on posterior face with 13 or 14 setae and three ventral setae, two of them stronger and larger than the rest. Third podomere subtrapezoidal, ~ 1.4× longer than wide, with ~ 4 posterodorsal and three slightly larger dorsal setae. Fourth podomere elongated, ~ 4.8× longer than wide, covered uniformly with microtrichiae, with ~ 17–19 setae arranged in six rows (only 15 visible in the Fig. 12), ventral and distal ones larger. Anterior claw mostly straight, ~ 2× as large as the posterior, posterior claw slender and slightly arched, frontal setae short, ~ 0.5× the length of the anterior claw. 12^th^ pair of legs with all podomeres with the anterior surface uniformly covered with micropubescence. First podomere subtriangular, ~ 1.4× wider than long, with ~ 3 setae on anterior face. Second podomere subtriangular, ~ 1.3× longer than wide, anterior face with ~ 12–14 setae of similar size and two ventral setae. Third podomere trapezoidal, ~ 1.1× wider than long, with ~ 4–6 anterior and 7–8 dorsal setae in two rows, dorsal setae larger. Fourth podomere subrectangular, ~ 1.8× longer than wide, with ~ 5 anterior setae, four or five posterior setae, two or three ventral setae, six or seven dorsal setae, the dorsal and posterior setae larger than the others, nearly twice as large. Fifth podomere elongated, ~ 4.4× longer than wide, with ~ 22-24 setae arranged in six longitudinal rows. Claws thick, arched, and subequal in size, frontal seta ~1/2 the length of the claws.

***Ventral surface***. Membranous and mostly granular in appearance, apart from the coxal sacs and styli. Sclerite near coxal sacs presents on every pair of legs except the first, of varying size with ~ 3–17 setae. Smallest near the second and last pair of legs. Last segment ventrally with micropubescence and ~ 18 setae. Last segment covering partially the base of the cerci ventrally. ***Coxal sacs***. (Fig. 13B). Fully developed only near the bases of legs 3–9, composed of two sclerites resembling the stomata of a plant, surface bearing micropubescence, distal sclerite relative to the base leg with ~ 4 setae, proximal sclerite with ~ 2. On pairs of legs 10 and 11, instead of a coxal sac, there is an elongated small sclerite with ~ 2-3 setae, and on leg 2, sclerite is circular with ~ 4-5 setae. ***Male organs*** (Fig. 13B) simple, two contiguous semicircular sclerites covered with micropubescence, each with a row of inconspicuous setae directed medially, plus a few scattered hairs outside these rows.

***Styli***. (Fig. 13D) Elongated with micropubescence, apically with a large conical seta along with another smaller seta. Larger seta 2.5× size of small one. ***Sense calicles***. (Fig. 13E) Pit margin surrounded by simple and some branched setae. Sensory seta with a wide base, inserted in the middle of the cavity, as long as the cerci in length. ***Cerci***. (Fig. 13A, C) Conical and with micropubescence, 2.4–3.2× longer than broad. Surface covered by 81 (60–99) setae, distal ones slightly longer than proximal. 20-25% basal portion glabrous but with micropubescence, apical 15% portion glabrous without any micropubescence. Apical setae 0.59–0.74× width of cerci. Smaller outer seta 0.33 as long as the apical seta.

###### Distribution.

Currently only reported in the municipalities of Girón and Lebrija in the department of Santander in Colombia.

###### Etymology.

The specific epithet lebrijana refers to the municipality of Lebrija, Santander, Colombia, where the type material was collected. The name is formed as an adjective derived from the toponym, in the feminine form, in agreement with the gender of the genus *Hanseniella*.

###### Remarks.

This species has also been detected in paprika crops [*Capsicum annuum* (L.) (Solanaceae)] (Montes-Rodríguez, pers. obs.).

##### Hanseniella chocoita sp. nov.

Taxon classificationAnimaliaSymphylaScutigerellidae

5EF2E4AA-DF3E-59F5-B67C-8047302166B1

https://zoobank.org/613DE0EB-7CD1-4862-BDD8-17CE19E8630E

**Figure 14.**
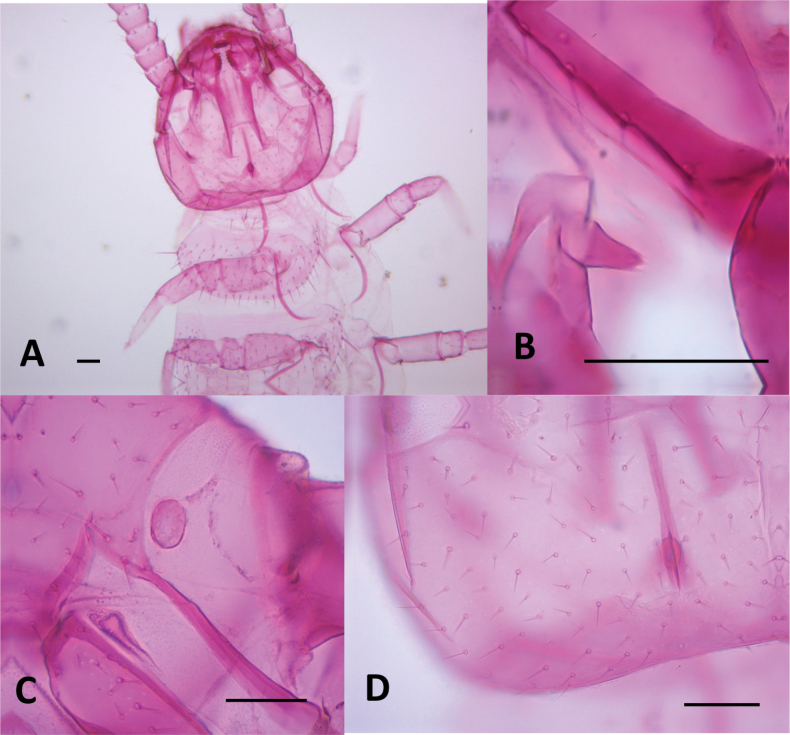
Head of *Hanseniella chocoita* sp. nov. **A**. Dorsal view of the head and first tergites (holotype); **B**. Conical maxillary palp (paratype); **C**. Side view of the head showing Tömösváry’s organ (paratype); **D**. Dorsal surface and regular setae of head (holotype). Scale bar: 50 µm.

**Figure 15.**
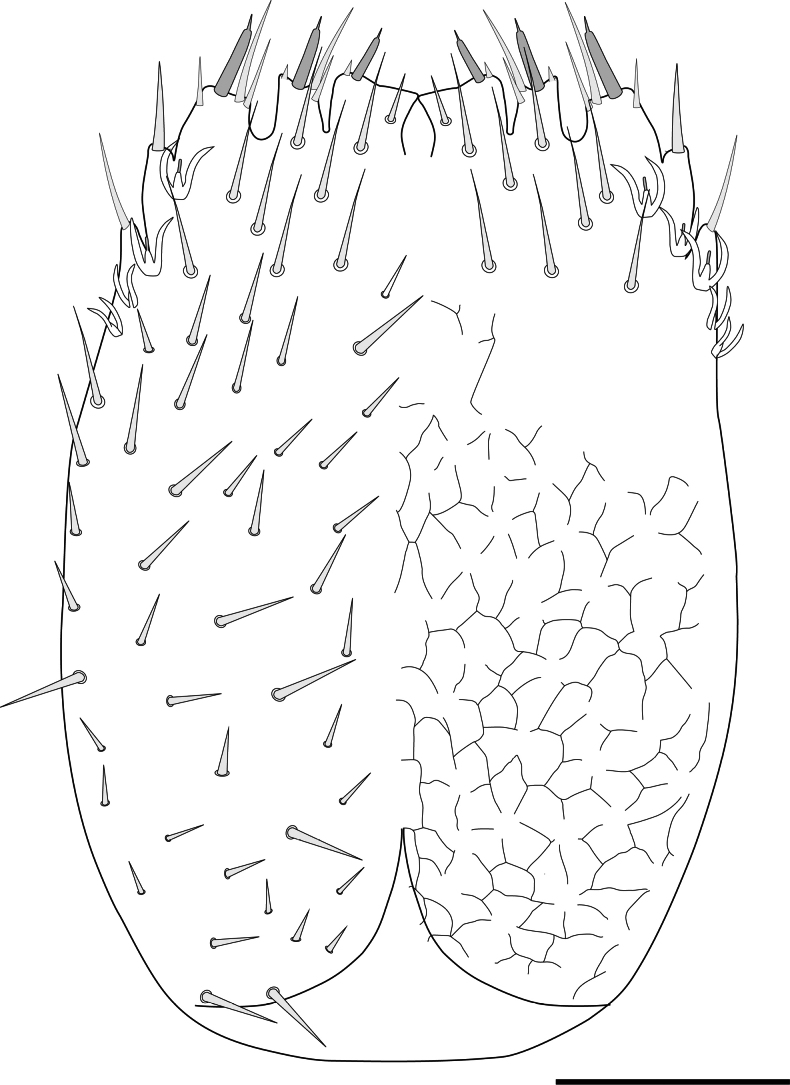
Anterior plates of the second maxillae of *Hanseniella chocoita* sp. nov. Left half with setae and right half with micro-sculpture of the cuticle. Scale bar: 50 µm

**Figure 16.**
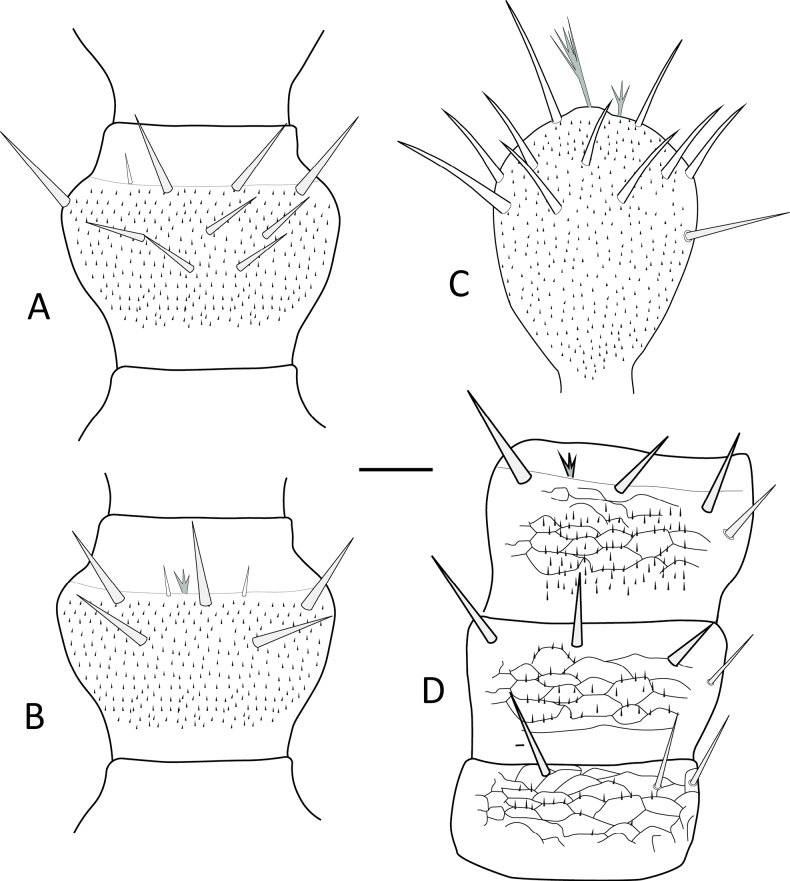
Antennae of *Hanseniella chocoita* sp. nov. **A, B**. Intermediate segments; **A**. Ventral; **B**. Dorsal; **C**. Last segment, dorsal view; **D**. Basal segments, dorsal view. Scale bar: 20 µm.

**Figure 17.**
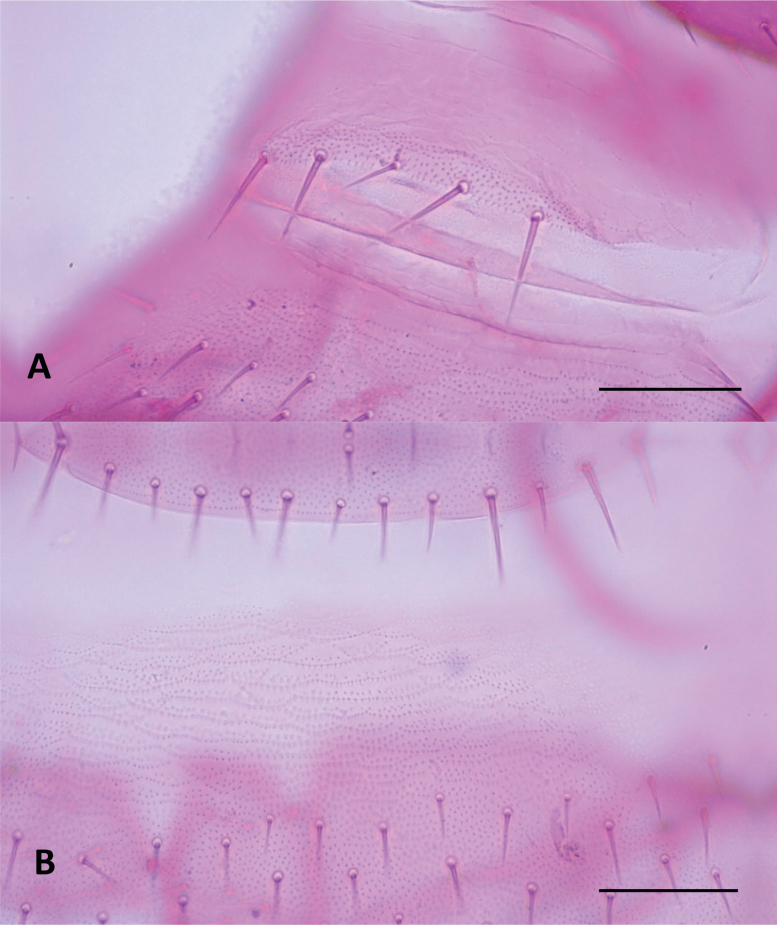
Tergites of *Hanseniella chocoita* sp. nov. **A**. First rudimentary tergite (paratype); **B**. Anterior area of the third tergite (holotype). Scale bars: 100 µm.

**Figure 18.**
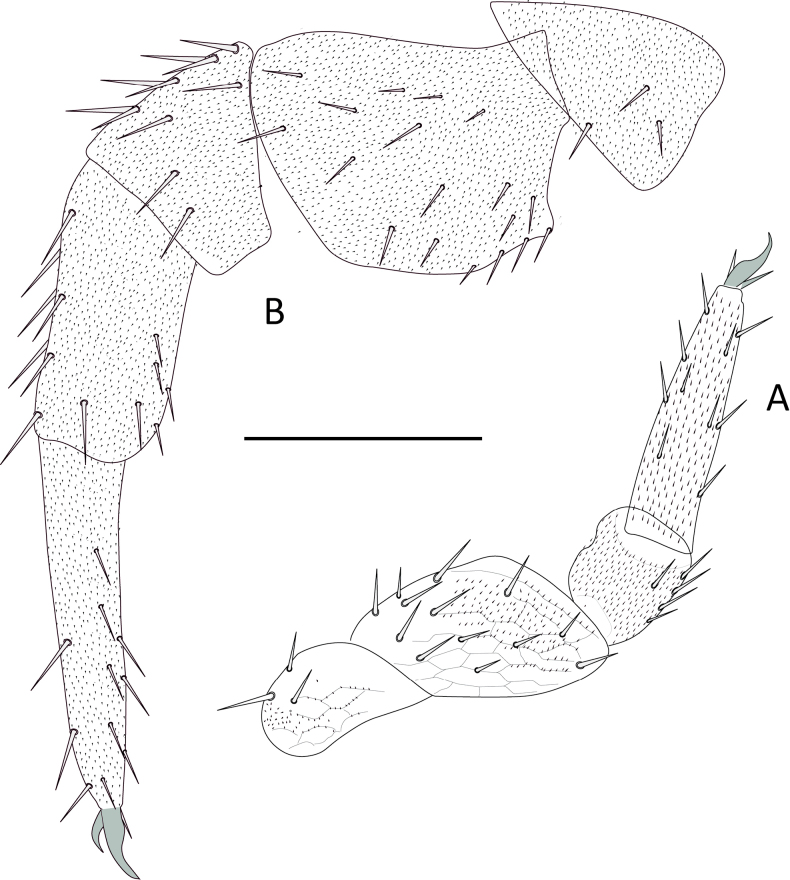
Legs of *Hanseniella chocoita* sp. nov. **A**. First pair of legs, posterior view; **B**. 12^th^ pair of leg, anterior view. Scale bar. 100 µm.

**Figure 19.**
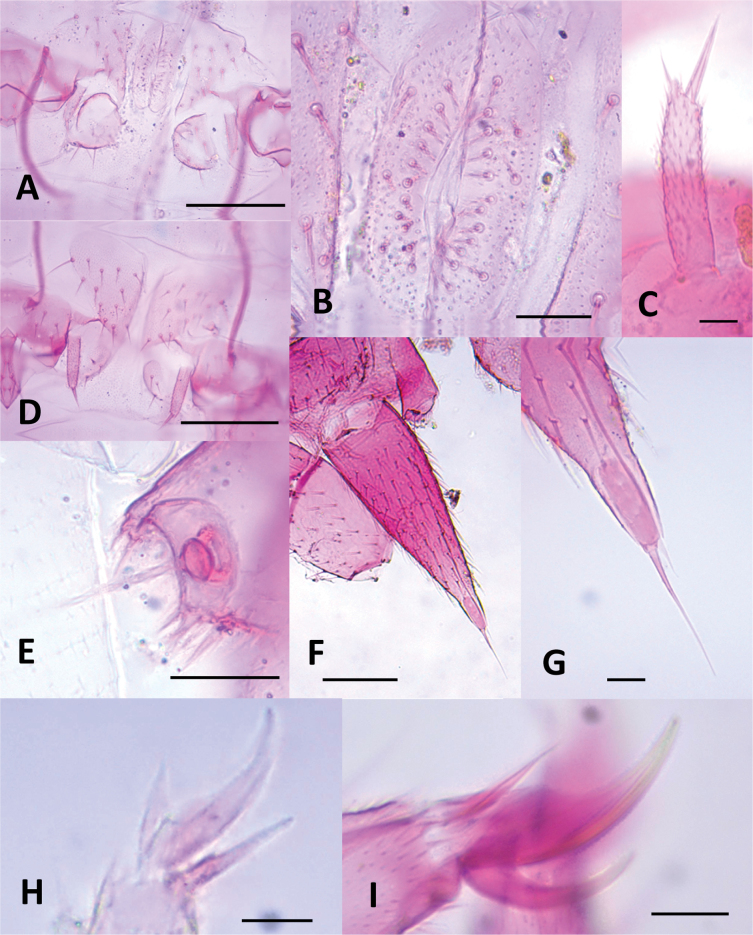
Cerci and ventral surface of *Hanseniella chocoita* sp. nov. **A**. Ventral view fourth pair of legs (holotype); **B**. Male organ (holotype); **C**. Styli (holotype); **D**. Ventral view tenth pair of legs (holotype); **E**. Sense calicles (holotype); **F**. Cerci (paratype); **G**. Apex of the cerci (paratype). **H, I**. Tarsal claws; **H**. First pair of legs; **I**. 12^th^ pair of legs (paratype). Scale bars: 100 µm (**A, D, F**); 20 µm (**B, E, G**); 10 µm (**C, H, I**).

Figs 14, 15, 16, 17, 18, 19

###### Type material.

***Holotype*** • male (CTNI-10574a), **Colombia**: Santander, Girón, Vereda Chocoita, manual collection in pineapple farming, 850 m a.s.l., 6.9867, -73.1628, 15-IX-2020. J. Montes-Rodríguez. ***Paratypes*** • 2 females, 1 male (CTNI-10574 b-d) same data as for holotype; • 2 females, 1 male (CTNI 10412 a-c) **Colombia**: Santander, Girón, Vereda Chocoita, manual collection in pineapple farming, 850 m a.s.l., 6.9867, -73.1628, 15-VIII-2020. J. Montes-Rodríguez.

###### Diagnosis.

*Hanseniella chocoita* sp. nov. shares the same pattern of macrochaetae on tergites 2–4, 6–7, and 9 with *H. aculeata* Jupeau, 1954, *H. afromontana* Scheller, 1954, *H. armigera* Scheller, 1961, *H. caldaria* (Hansen, 1903), *H. colombiana* Juberthie-Jupeau & Réveillet, 1997, *H. conisetosa* Scheller, 1971, *H. echinata* Adam & Burtel, 1956, *H. ghanensis* Belfield, 1988, *H. guimaraensis* Scheller, 2007, *H. hortulana* Scheller, 1971, *H. incompta* Scheller, 1971, *H. ivorensis* Juberthie-Jupeau & Kehe, 1978, *H. lebrijana* sp. nov. Montes, Parra-Gómez, Holguín & Marchant, 2025, *H. lucifuga* Scheller, 1961, *H. milloti* Aubrey & Masson, 1953, *H. modesta* Aubrey & Masson, 1953, *H. montana* Scheller, 1971, *H. orientalis* (Hansen, 1903), *H. remyi* Aubrey & Masson, 1953, *H. similis* Scheller, 1961 and *H. unguiculata* (Hansen, 1903). It differs from them by the following combination of characters: body length 3.4–4.4; distinct central rod on head; first maxillary palp with the apex divided into three tips, central tip broader than flanking ones; surface of antennomeres 4 and 5 with scaly pattern mixed with micropubescence; antennae with three whorls maximum, simple setae of varying size and small trifurcate organs; apical antennomere with usual stalk organ, plus a smaller one; first rudimentary tergite with four or five setae; posterior margin of tergite 2 slightly convex, in tergite 3 mostly straight, in tergites 13 or 14 sightly concave; anterior margin of all tergites glabrous, with pattern of transverse arched mixed with micropubescence; sclerites near coxal sacs with simple setae; first pair of legs with posterior claw straight and subulate, and frontal setae short and lanceolate; 12^th^ pair of legs with anterior claw with proximal half thick, posterior portion moderately arched and relatively broad, tip acuminate; styli with two setae, with smaller setae arising from a tiny conical protuberance at the apex of styli; cerci setose, bearing simple setae and with a significant basal portion glabrous and with micropubescence. It can also be differentiated from the most similar species by markedly different size and shape of claws of the 12^th^ pair of legs in *H. chocoita* sp. nov., while similar in size in *H. aculeata*. A greater number of setae on the cerci (75–106), while *H. montana* has fewer than 20. The length of the cerci of *H. hortulana* and *H. armigera* is 4× longer than wide, while in *H. chocoita* sp. nov. it is less than 3.17. *Hanseniella similis* in the equiangular trapezoidal shape of the third tergite, whereas in *H. chocoita* sp. nov. it is subrectangular. It differs from *Hanseniella milloti* in the shape of its cerci, which is relatively shorter and has fewer and longer setae than *H. chocoita* sp. nov. and differs from *H. incompta* in the tip of the maxillary palp, which is divided in *H. chocoita* sp. nov. and continuous in *H. incompta*.

###### Description.

Length of body without antennae and cerci 3.4 (3.4–4.4) mm, antenna 1.15–1.37 (1.15–1.71). ***Head***. Slightly longer than wider, 1.01 (1.01–1.17) × broader than long, frontal margin convex, lateral margin at point of articulation smooth, posterior margin straight with rounded posterolateral angles (Fig. 14A). Central rod ovoid posteriorly (Fig. 14D). Dorsal surface smooth without micropubescence or microsculpture, except for the anterior area of the head between the antennae, which has a scale-like cuticle. Dorsal surface covered by straight setae not significantly different, few large setae ~ 2.0× longer than normal setae and 0.50× the width of the first antennomer, arranged in 3+3 anteriorly of antennal insertion, four on the anterior margin of the head, four near the anterior margin and three laterally on each side behind the rounded Tömösváry’s organ. Area around Tömösváry’s organ granular in texture (Fig. 14C). Each anterior plate of the second maxillae with two proximal setae. External-distal corner of these plates with four or five sets of sensilla with typical chandelier shape decreasing in size proximally and two elongated setae inserted on conic protuberances. Three terminal protuberances with 1–3 setae plus a single large distal sensillum, the two medial protuberances with a contiguous tiny tooth (Fig. 15). First maxillary palp conical with the apex divided into three tips, central tip broader than flanking ones (Fig. 14B).

***Antennae***. (Fig. 16). Long, 0.33–0.40× the length of the body, composed of 20–24 antennomers. Surface with scaly pattern mixed with micropubescence present from first to 4–5 antennomers, gradually disappearing as micropubescence become more abundant; from segment 6 onwards, pattern no longer visible and micropubescence uniform. First antennomere 1.59× wider than longer, with a whorl of setae initially with three outer setae, from the second antennomere onwards with 7–10 setae. Second antennomere 1.84× wider than longer. Second whorl with two types of setae: (i) Small inner setae, three on first antennomere and gradually decreasing in number until disappearing on distal antennomers. (ii) larger setae appear on the intermediate segments with three setae and gradually increasing up to ten setae towards the last antennomeres. Third whorl starts in the middle segments with one seta and ends in the last segments with two. An additional intermediate seta between whorls 2 and 3 present on the penultimate and antepenultimate antennomers. Apical antennomere spherical, apex bearing a large sensory organ borne from a small protuberance, composed of a central stalk which yields five slightly longer spiniform processes. One additional similar organ smaller and without protuberance.

***Tergites***. (Fig. 17, Table 8) Cuticle with the area anterior to setae with pattern of transverse arches mixed with micropubescence. First tergite rudimentary, with only four or five central setae. From third tergite onwards: 0.33–0.5 anterior surface without setae, pattern of transverse arches imperceptible in the area with setae. Four types of setae of different length present on tergites: (i) small and similar in size, covering the tergal surface; (ii) ~ 2× as large as the anterior type, sparsely present on the posterior margin; (iii) ~ 1.5× larger than the large marginal ones, located on the posterolateral corners except on tergites 1, 14, and 15; (iv) Macrochaetae in segments 2–4, 6, 7, and 9, ~ 1.5× larger than the anterior type.

**Table 8.** Description of tergites and number of tergal setae in *Hanseniella chocoita* sp. nov. holotype.

Tergite	Shape	Posterior margin	Total number of setae	Setae in the posterior margin
2	Semicircular	Slightly convex	83	31
3	Semicircular	Almost straight	111	31
4	Subrectangular	Slightly concave	122	34
5	Subrectangular	Slightly concave	90	36
6	Subrectangular	Slightly concave	192	43
7	Subrectangular	Slightly concave	154	43
8	Subrectangular	Slightly concave	105	39
9	Subrectangular	Slightly concave	207	48
10	Subrectangular	Slightly concave	152	44
11	Subrectangular	Slightly concave	107	36
12	Subrectangular	Slightly concave	189	50
13	Subrectangular	Slightly concave	128	39
14	Subrectangular	Slightly concave	148	37
15	Subquadrate	Convex between cerci	45	15

**Legs**. (Figs 18, 19H, I) First pair of legs composed of four segments. First podomere subrectangular, ~ 1.3× longer than wide, with poorly defined scaly pattern, scattered micropubescence and ~ 3 posteroventral setae. Second podomere subrectangular, ~ 1.4× longer than wide, most of the posterior surface with a scaly pattern and scattered micropubescence that becomes denser in the distal area, with ~ 9 setae on posterior face and four ventral setae. Third podomere subrectangular, 1.15× longer than wide, weak hexagonal pattern and micropubescence, with ~ 1 posterodorsal and four dorsal setae. Fourth podomere elongated, ~ 3.8× longer than wide, covered uniformly with micropubescence, with ~ 13 setae arranged in five rows (only ten visible in Fig. 18). Anterior claw basally thick, medially arched, ~ 2× as large as the posterior, posterior claw straight and subulate, frontal setae short and lanceolate, ~ 0.5 length of the anterior claw.

12^th^ pair of legs with all podomeres bearing micropubescence in anterior view. First podomere subtriangular, ~ 1.5× wider than long, with three or four anteroventral setae. Second podomere subrectangular, ~ 1.3× longer than wide, anterior face with ~ 14 setae of similar size and four ventral setae. Third podomere trapezoidal, ~ 1.03× longer than wide, with ~ four on anterior face and seven dorsal setae in two rows. Fourth podomere subrectangular, ~ 2× longer than wide, with ~ 4 setae on anterior face, seven dorsal setae in two rows, two or three ventral setae and five setae on posterior face (not visible in Fig. 18), the posterior and dorsal setae larger than the others. Fifth podomere elongated, ~ 4.4× longer than wide, with ~ 21 setae arranged in six longitudinal rows (only 11 visible in Fig. 18). Claws different in size, proximal half of anterior claw thicker than the distal half, posterior portion moderately arched and relatively broad, posterior claw arched and slender than anterior claw, frontal seta lanceolate, about half the length of the anterior claw. ***Ventral surface***. (Fig. 19A, D) Membranous and mostly granular in appearance, apart from the coxal sacs and styli. Sclerites near coxal sacs presents on every pair of legs except the first. Smallest near the second and last pair of legs, elongated with ~ 3–5 setae. Rest of sclerites subtriangular with ~ 8–33 setae. Last segment ventrally with micropubescence and transverse arch pattern, with ~ 18 setae. Last segment covering partially the base of the cerci ventrally. ***Coxal sacs***. (Fig. 19A, D). Fully developed only near the bases of legs 3–9, composed of two semicircular sclerites, surface bearing micropubescence, distal sclerite relative to the base leg with ~ 3–5 setae, proximal sclerite with two or three. Leg pairs 2, 10, and 11 with sclerites bearing ~ 2–5 setae. Sclerites of leg pair 2 oval-shaped, on pairs 10 and 11 subtriangular. ***Male organs***. (Fig. 19A, B) Simple, two contiguous semicircular sclerites covered with micropubescence, each with a row of conical setae directed medially and three or four outer setae. ***Styli***. (Fig. 19C, D) Elongated with micropubescence, apically with a large conical seta along with another smaller seta. The small seta arises from a tiny conical protuberance at the apex of the styli. Larger seta 2.5× the size of the small one. ***Sense calicles***. (Fig. 19E) Pit margin surrounded by simple and some branched setae. Sensory seta with a wide base, inserted in the middle of the cavity, as long as the cerci in length. ***Cerci***. (Fig. 19F, G) Conical and with micropubescence, 3.08–3.17 longer than broad. Surface covered by 75–76 (75–116) setae, distal ones slightly longer than proximal. 15% basal portion glabrous, apical 20% portion also glabrous, but with only 15% without any micropubescence. Apical setae 0.65–0.66× the width of cerci. Accompanying seta 0.3× the apical seta.

###### Distribution.

This species has only been collected in the type locality in the Chocoita district of the municipality of Girón in the department of Santander in Colombia.

###### Etymology.

The specific epithet chocoita refers to the locality, where the type material was collected. Feminine, in agreement with the gender of the genus *Hanseniella*.

###### Remarks.

The correct identification of *Hanseniella chocoita* sp. nov. requires careful examination under microscope due to subtle differences in the chaetotaxy of the antennae with *Hanseniella cf. unguiculata*. *Hanseniella chocoita* sp. nov. was collected only during the rainy season when the soil was more humid.

##### Hanseniella

Taxon classificationAnimaliaSymphylaScutigerellidae

sp. 5

E0676537-2D41-51B2-9B81-DC66F9329E74

###### Material studied.

• 1 female, **Colombia**: Santander, Girón, Vereda Chocoita, manual collection in pineapple farming, 850 m a.s.l., 6.9867, -73.1628, 15-IX-2020. J. Montes-Rodríguez, GICho01-1 (field code).

###### Distribution.

This species has only been collected in the Chocoita district of the municipality of Girón in the department of Santander in Colombia.

###### Remarks.

**Figure 20.**
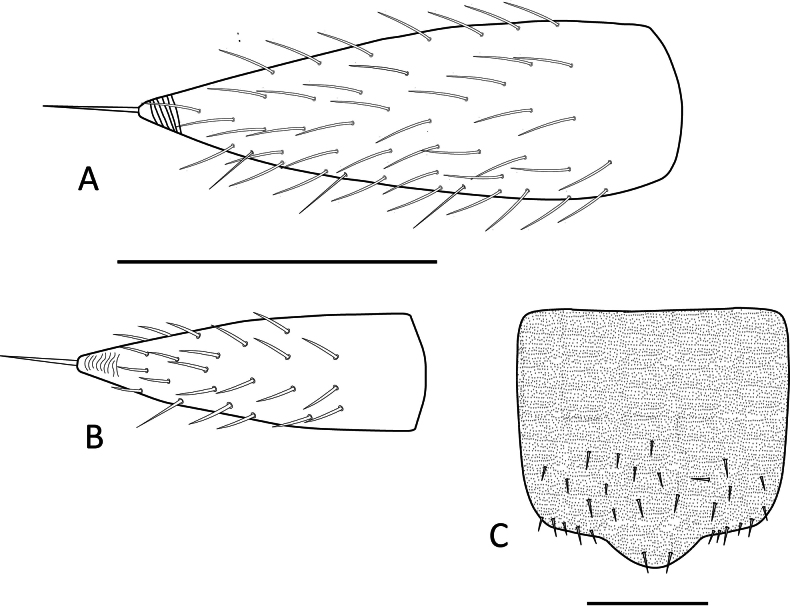
Morphological characteristics of symphylans in pineapples from Santander, Colombia. **A**. Dorsal view of right cerci from *Symphylella* sp. 1; **B**. Dorsal view of right cerci from *Symphylella* sp. 2; **C**. Dorsal view of last tergite from *Hanseniella* sp. 5; Scale bars: 100 µm.

This is the first species of the genus *Hanseniella* reported in Neotropics with the posterior margin of the last tergite with a median projection or expansion between the cerci (Fig. 20C). This morphological characteristic is also present in *H. brachycerca*, described in New Zealand by Adam and Burtel (1956). The taxonomic key proposed by Scheller and Adis (2002) for identifying Neotropical genera uses this characteristic to differentiate between the genera *Hanseniella* and *Scopoliella*, assuming that all *Hanseniella* species in the Neotropics had a straight or almost straight posterior margin on the last tergite. With the presence of *Hanseniella* sp. 5, this key was adjusted by incorporating other characteristics of *Scopoliella* (See below).

#### Genus *Symphylella* Silvestri, 1902

##### Symphylella

Taxon classificationAnimaliaSymphylaScolopendrellidae

sp. 1

4FE47C71-F8F8-50ED-932C-D65E43422BAD

###### Material studied.

• 1 female, 1 male (CTNI- 10414- 10415) **Colombia**: Santander, Lebrija, Vereda la Puente, manual collection in pineapple farming, 1297 m a.s.l., 7.0790, -73.2033, 15-XI-2019, J. Montes-Rodríguez; • 1 male **Colombia**: Santander, Lebrija, Vereda la Puente, manual collection in pineapple farming, 1297 m a.s.l., 7.0790, -73.2033, 15-IX-2019. J. M. Montes-Rodríguez, LELaPue-10 (field code); • 2 females **Colombia**: Santander, Lebrija, Vereda La Aguada de Ceferino, manual collection in pineapple farming, 975 m a.s.l., 7.1978, -73.1714, 15-X-2019, J. Montes-Rodríguez, LELaAgu01- 11-12 (field code).

###### Distribution.

This species has only been collected in the municipality of Lebrija in the department of Santander in Colombia.

###### Remarks.

Among the species reported in the Neotropics, this could be most closely related to *Symphylella neotropica* Hansen, 1903. The similarities are in the head, specifically in the central rod, with the anterior part conspicuous but narrow and the frontal branches are thin. The conformation of the antennal setae is also similar to *S. neotropica*, with the first whorl having larger setae than the second.

The original description describes the cerci as having a moderate number of setae, with six or seven protruding on the lower half of the outer surface, while the remaining setae are inclined. In *Symphylella* sp. 1, there are a similar number of setae, but the erect setae are not significantly different in length from the rest and tend to be in the distal half of the cerci (Fig. 20A). Furthermore, the shape of tergites II and III resembles those described by Hansen (1903) for *S. neotropica*. However, *Symphylella* sp. 1 has only short setae in contrast of the several long marginal setae present on tergite 3 (= scutum 2 according to the original interpretation of Hansen [1903]) in *S. neotropica*.

##### Symphylella

Taxon classificationAnimaliaSymphylaScolopendrellidae

sp. 2

3290447E-788F-513A-8FD6-81565C4A3501

###### Material studied.

• 2 females, 1 male, **Colombia**: Santander, Girón, Vereda Chocoita, manual collection in pineapple farming, 850 m a.s.l., 6.9867, -73.1628, 15-IX-2020, J. Montes-Rodríguez, GICho01-15-17 (field code).

###### Distribution.

This species has only been collected in the Chocoita district of the municipality of Girón in the department of Santander in Colombia.

###### Remarks.

This species does not resemble any of the species of the genus described in the Neotropics. The cerci has a very distinct dorsolateral seta and a row of three erect setae lateroventrally (Fig. 20B).

### Key to genera of Central and South America Scutigerellidae (modified from Scheller and Adis 2002)

**Table:** 

1	With deep cavity beneath middle of posterior margin of last tergite	*** Scutigerella ***
–	Without cavity beneath middle of posterior margin of last tergite	**2**
2	Posterior and posterolateral margins of tergites 2–14 not crenate, conical setae on cerci absent	*** Hanseniella ***
–	Posterior and posterolateral margins of tergites 2–14 always crenate, conical setae on cerci present	*** Scopoliella ***

### Key to adults of South American *Hanseniella*

**Table:** 

1	Pattern of lateral macrochaetae on the tergites 2–4, 6, 7, and 9	**2**
–	Lateral macrochaetae without arrangement 2–4, 6, 7, and 9	**8**
2	Claws on pair of legs 12 of similar size and shape	**3**
–	Claws on pair of legs 12 of different sizes and shapes	**5**
3	Long setae on the trochanter of leg I	***H. orientalis***
–	Without long setae on the trochanter of leg I	**4**
4	Without central rod. With very short femur XII, ~2× as wide as long	***H. guimaraensis***
–	With central rod. With femur not so short, approximately as wide as long.	***H. lebrijana* sp. nov**.
5	Two setae on the first tergite	***H. colombiana***
–	Four or five setae on the first tergite	**6**
6	Second whorl of setae the same size as the central or primary whorl, and the ventral and dorsal setae of the antennae the same size	***H. unguiculata***
–	Ventral and dorsal setae on the antennae of different sizes	**7**
7	Antennae with relatively small setae on the first whorl	***H. caldaria***
–	Antennae with longer setae on the first whorl	***H. chocoita* sp. nov**.
8	Pattern of lateral macrochaetae on the tergites 2–4, 6–7, 9–10 and 12–13	***H. arborea***
–	Lateral macrochaetae without arrangement 2–4, 6–7, 9–10 and 12–13	**9**
9	Posterior margin of tergite XV with distinct medial projection	***H.* sp. 5**
–	Posterior margin of tergite XV straight, without medial projection	**10**
10	Lateral macrochaeta on tergite V	***H. paoletti***
–	Lateral macrochaeta absent on tergite V	**11**
11	9 to 11 setae on the first tergite	***H. chilensis***
–	Two setae on the first tergite	**12**
12	Lateral macrochaeta on tergite 13, sometimes on 12	***H. guerreroi***
–	Without lateral macrochaeta on tergite 13	***H. longisetis***

## Discussion

This study fundamentally improves the understanding of symphylan diversity within Colombian pineapple agroecosystems. Our work reveals a far more complex diversity than the two species previously reported: *Scutigerella immaculata* and *Hanseniella colombiana* (Agredo et al. 1988; García and Martínez 1991; Juberthie-Jupeau and Réveillet 1997). By identifying six species not previously reported for Colombia across the genera *Hanseniella* and *Symphylella*, we provide a new baseline for symphylan taxonomy in the region. These findings confirm that the Andean region in Colombia, a center of intensive agriculture, represents a major blind spot in Neotropical soil biodiversity studies, emphasizing the severe under-sampling of symphylans in this region. Furthermore, the reliance on baited traps may have biased our collection towards more active, surface-foraging species, potentially under-representing deeper-dwelling taxa. Future biodiversity surveys employing a wider range of techniques, such as Berlese funnel extraction, will likely reveal the presence of other genera and even more undescribed species in the region.

### An integrative approach for resolving symphylan taxonomy

A central finding of our study is the value of an integrative taxonomic framework to interpret morphological variation and delimit species boundaries in a challenging group like Symphyla. The phylogenetic analysis of the COI gene, corroborated by a clear barcoding gap in the K2P genetic distances, provided a robust hypothesis of species delimitation. The maximum intra-clade divergence was consistently low (≤ 1.0%), whereas the minimum inter-clade divergence was an order of magnitude higher (≥ 17%), providing strong quantitative support for the separation of the four major clades.

This molecular framework was essential for reinterpreting morphological characters that were initially thought to distinguish species. For instance, individuals assigned to *Hanseniella* sp. 3 and sp. 4, which differ in the number of setae on the first tergite, were resolved within a single, genetically cohesive lineage (Clade 2). This indicates that this difference in setation represents intraspecific rather than interspecific variation, within what is now described as *Hanseniella chocoita* sp. nov. Similarly, *Symphylella* sp. 1 and sp. 3, separated by subtle chaetotaxic differences, were recovered as closely related sister haplotypes (Clade 4), suggesting they belong to a single species. Rather than revealing conflict, the molecular data provided the necessary context to correctly interpret morphological variability, thereby preventing taxonomic oversplitting that can arise when intraspecific variation in labile traits is misinterpreted as evidence of interspecific differentiation.

### Characters in Symphyla

The taxonomy of Symphyla present several difficulties, largely due to the scarcity of detailed morphological descriptions in historical literature. Structures such as the first maxillary palp, the second maxilla, antennal organs, the apical antennomere (often missing due to abrasion or natural causes), the first rudimentary tergite setae and shape, ventral surface, coxal sacs, male organs, and detailed descriptions of the claws of the first and twelfth pairs of legs (usually only the anterior claws are described in detail), are frequently incompletely described or entirely overlooked (e.g., Hansen 1903; Aubry and Masson 1953; Edwards 1959; Juberthie-Jupeau 1962). This lack of comprehensive characterization reduces the set of comparable features available across species, making delimitation increasingly problematic when working with older descriptions. Consequently, the redescription of foundational taxa, particularly those originally described by Hansen (1903), remains an urgent task for the few symphylan taxonomists across the world in order to improve and facilitate diagnoses.

In this context, our findings provide insight into which morphological differences are biologically meaningful, as a very low genetic differentiation between some morphologically distinct forms suggests that these variations likely reflect phenotypic plasticity or simple polymorphism within species, rather than reliable interspecific characters. Thus, identifying the characteristics that consistently separate species within the genera *Hanseniella* and *Symphylella* is the critical next step in advancing Neotropical symphylan taxonomy. Among the most useful traits, the setae on the first tergite proved particularly informative, although their variation is better interpreted in terms of ranges rather than fixed values. In *Hanseniella*, we observed two main conditions: two setae (occasionally 3), and four or five setae; in *Symphylella*, we identified configurations of 3+3 or 4+4–4+5. Additional characters with considerable potential lie in the antennal chaetotaxy. To separate *Hanseniella* species, the presence and lengths of the second and third whorls of setae on the intermediate antennal segments have generally been used successfully. In our case, all *Hanseniella* species finally determined had a clearly distinguishable pattern on the antennae setae that was species-specific.

However, a major limitation of this study in assessing intraspecific variation and the utility of morphological characters was the limited number of individuals captured for most species. Of the 786 adults collected across the six species ultimately identified, four species were represented by fewer than seven individuals. Among these, some specimens were lost or damaged during mounting, and others were impossible to examine. This scarcity and loss of specimens also prevented the successful sequencing of all morphospecies (i.e., *Hanseniella* sp. 5 and *Symphylella* sp. 2), thereby restricting the ability to test the validity of these.

On the other hand, although the slide mounting method for insects of the superfamily Coccoidea yields good results in most cases, it should be used with caution, and a more specific process should be standardized for symphylans. While scanning electron microscopy has often been applied in recent times to examine and describe symphylans (e.g., Domínguez and Vandenspiegel 2012; Salazar-Moncada et al. 2015; Parra-Gómez et al. 2024; Porta et al. 2024), an accessible and reliable slide-mounting protocol remains essential, as it would enable a broader community to describe new species and report new records, especially in institutions where this technology is unavailable or prohibitively expensive.

### *Hanseniella cf. unguiculata*: a widespread and dominant agricultural pest

From an applied perspective, our findings have direct implications for pest management. The data reveal that the symphylan pest complex is not an intractable assemblage of numerous species, but is overwhelmingly dominated by a single, widespread, and abundant species: *Hanseniella cf. unguiculata*. This finding strongly suggests that this species is the principal agent responsible for the direct root damage, nutrient uptake inhibition, and yield loss characteristic of symphylan infestation in pineapple (Rohrbach and Johnson 2003). Furthermore, it is likely the main facilitator of secondary infections by soil-borne pathogens like *Fusarium* and *Phytophthora*, by creating wounds that serve as entry point for these organisms, a critical issue for pineapple crops (Saavedra 1990). In contrast, the other species were found in much lower abundances and had more restricted distributions, suggesting they may be secondary pests or incidental members of the soil arthropod fauna.

It is important to note that the COI sequences of the specimens reported by Salazar-Moncada et al. (2015) as members of Scutigerellidae show minimal genetic divergence from our COI sequences of *Hanseniella cf. unguiculata*. This close similarity indicates that both sets of specimens represent the same taxon, thereby extending the known distribution and host range of *H. cf. unguiculata*. In their study, the species was identified as a pest of flowers in the Colombian departments of Antioquia and Cundinamarca, at elevations of 2180 and 2548 m a.s.l., respectively. In combination with our records, the available data provides independent evidence of polyphagous habits, a wide altitudinal distribution, and an apparent remarkable adaptability. Subsequent work further demonstrated that populations of this species are susceptible to entomopathogenic fungi, confirming the potential of biological control agents as a management strategy for this species (Salazar-Moncada et al. 2020). This approach could be adapted and validated for use in pineapple cropping systems. On the other hand, it is worth mentioning that Salazar-Moncada et al. (2015) reported that their COI haplotypes are identical to sequences from specimens collected in Cameroon and submitted to Barcoding of Life Data Systems (BOLD), suggesting a broader geographic distribution. A new search in BOLD retrieved a specimen from Thailand, with a COI sequence 100% identical to ours. Interestingly, however, we were unable to locate the Cameroonian sequences in BOLD as mentioned by Salazar-Moncada et al. (2015).

Overall, by clarifying the taxonomic identity of the primary pest species and resolving the long-standing confusion between *Scutigerella immaculata* and *Hanseniella colombiana*, our work removes a critical impediment that has historically limited the development of precise, evidence-based management strategies in this pest group in Colombia.

Furthermore, our species delimitations are based on a single mitochondrial marker, a standard approach in foundational taxonomic and biodiversity studies. While it is well-established that the evolutionary history of a single gene can sometimes differ from that of the species (Maddison 1997; Funk and Omland 2003; Edwards 2009), the evidence in this study indicates that the COI gene was useful to support the species identification within this symphylan assemblage. This conclusion is supported by the clear barcoding gap demonstrated in our analysis: the maximum intra-clade divergence (≤ 1.0%) is an order of magnitude lower than the minimum inter-clade divergence (≥ 17%). This finding aligns with other studies on taxonomically challenging arthropod groups, including myriapods. For instance, the COI gene has been shown to successfully identify more than 95% of a total of 122 species from Germany (Spelda et al. 2011). In groups where morphology is conserved or ambiguous, COI barcoding has become a valuable tool for delimiting species, identifying cryptic lineages, and guiding taxonomic revisions (Hebert et al. 2003; Foottit et al. 2014). Given our results, this study provides a validated framework for using COI as a primary marker for the rapid identification of symphylan species in the Neotropics. Nonetheless, integrating multi-locus datasets that include nuclear genes will be an important next step for resolving deeper phylogenetic relationships and reassessing mitochondrial species delimitation within this group.

## Supplementary Material

XML Treatment for Hanseniella cf. unguiculata

XML Treatment for Hanseniella lebrijana

XML Treatment for Hanseniella chocoita

XML Treatment for Hanseniella

XML Treatment for Symphylella

XML Treatment for Symphylella

## Additional information

### Use of AI

No use of AI was reported.

### Author ORCIDs

José Mauricio Montes-Rodríguez https://orcid.org/0000-0002-9281-2991

Claudia M. Holguin https://orcid.org/0000-0002-6498-3195

Antonio Parra-Gómez https://orcid.org/0000-0002-4144-6610

Sergio Marchant https://orcid.org/0000-0002-7362-3626

### Data availability

All of the data that support the findings of this study are available in the main text.
